# Effect of online hemodiafiltration compared with hemodialysis on quality of life in patients with ESRD: A systematic review and meta-analysis of randomized trials

**DOI:** 10.1371/journal.pone.0205037

**Published:** 2018-10-18

**Authors:** Tatsuya Suwabe, Francisco J. Barrera-Flores, Rene Rodriguez-Gutierrez, Yoshifumi Ubara, Kenmei Takaichi

**Affiliations:** 1 Division of Nephrology and Hypertension, Mayo Clinic, Rochester, Minnesota, United States of America; 2 Division of Nephrology, Toranomon Hospital, Tokyo, Japan; 3 Division of Endocrinology, Medical School and University Hospital “Dr. Jose E. Gonzalez”, Universidad Autonoma de Nuevo Leon, Monterrey, Mexico; 4 Plataforma INVEST Medicina UANL KER Unit Mayo Clinic, Medical School and University Hospital “Dr. Jose E. Gonzalez”, Universidad Autonoma de Nuevo Leon, Monterrey, Mexico; UNSW Sydney, AUSTRALIA

## Abstract

**Background:**

End-stage renal disease (ESRD) is related to high morbidity, mortality, and impaired health-related quality of life. While hemodialysis (HD) is the current life-saving standard of treatment for patients with ESRD, their quality of life (QoL) remains far from desirable. Online HDF (OL-HDF), due to its convenience, could improve the QoL of patients with ESRD, however, this remains uncertain.

**Objective:**

We aimed to assess the body of evidence of OL-HDF compared to HD regarding QoL in patients with ESRD.

**Methods:**

We comprehensively searched in multiple data bases from their inception to February 2018. Reviewers working independently and in duplicate appraised the quality and included randomized controlled trials (RCTs) that evaluated, in patients with ESRD and HD or OL-HDF, QoL (Short Form Health Survey with 36 questions (SF-36) with physical component score (PCS) and mental component score (MCS) as well as scores about social activity, fatigue, and emotion). A meta-analysis of each outcome of interest was performed using a random-effects model.

**Results:**

Six moderate quality RCTs met the inclusion criteria. Meta-analysis of 4 RCTs including a total of 1,209 patients showed that OL-HDF was associated with a lower yet non-significant score of PCS: MD (mean difference) -0.77 (95% CI -1.94 to 0.41, p = 0.20), and MCS: MD -1.25 (95% CI -3.10 to 0.59, p = 0.18); indicating a poorer QoL in patients on OL-HDF. Meta-analysis of 4 RCTs including a total of 845 patients showed OL-HDF was associated with a significant increase in the score of social activity compared to HD: SMD (standardized mean difference): 1.95 (95% CI 0.05 to 3.86, p = 0.04), indicating a better QoL in patients on OL-HDF; but regarding fatigue and emotion, there was no significant improvement when compared to HD by meta-analysis of 3 RCTs (133 patients).

**Conclusions:**

The body of evidence suggests that OL-HDF does not improve QoL in patients with ESRD when compared to HD.

## Introduction

For any healthcare system, the increasing number of patients with end-stage renal disease (ESRD) is a major concern and health priority. Globally, over 2 million people receive treatment with dialysis or a kidney transplant [1], and more than 700,000 patients per year in the United States are affected by ESRD. Hemodialysis (HD) is a life-saving and the current standard of treatment for patients with ESRD. However, survival rate and quality of life (QoL) of patients in this scenario is far from desirable.

Previous studies suggest that convective modalities such as hemofiltration (HF) and hemodiafiltration (HDF) are associated with better removal of both small and middle molecules as well as greater hemodynamic stability, improved survival, and better QoL of patients with ESRD, compared with standard HD [2–7]. HDF combines diffusive and convective clearance of uremic solutes. The utilization of online HDF (OL-HDF), where replacement fluid is prepared by further purifying dialysate fluid instead of manufacturer-provided solutions has made HDF more practical and more cost-effective. It is believed that high-volume OL-HDF could potentially improve symptomatology, reduce morbidity, and may even improve survival [8]. The number of worldwide patients receiving this modality has doubled between 2004 and 2010, and has reached 80,000 [9]. OL-HDF was officially approved for the treatment of ESRD patients and for reimbursement by the Japanese health insurance system in 2012. Thereafter, the number of OL-HDF patients in this country has been dramatically increasing [10].

Despite the data that suggests that OL-HDF can improve the patients symptoms, morbidity, and survival in patients with ESRD, recent randomized clinical trials (RCTs) have failed to demonstrate consistent results regarding improvement in QoL; hence, the magnitude of this benefit, if present, remains uncertain [11–16]. To our knowledge, there has been only one evidence synthesis that quantifies the QoL of patients on therapy with OL-HDF compared with standard HD, nevertheless this study didn’t include any RCTs after 2007 [17]. Therefore, we aimed to assess the body of evidence of the effectiveness of OL-HDF regarding the QoL in patients with ESRD when compared to standard HD.

## Methods

This study was conducted following guidance provided by the Cochrane Handbook for systematic reviews[18]; and it is reported in accordance to the recommendations set by the Preferred Reporting Items for Systematic Reviews and Meta-Analyses (PRISMA) work group. The protocol of this study has been deposited in protocols.io. and the identifier (DOI) of this study has been assigned as dx.doi.org/10.17504/protocols.io.rhnd35e.

### Eligibility criteria

We included RCTs that compared OL-HDF against normal HD (high- or low-flux) in adult patients with ESRD. We excluded RCTs that enrolled patients receiving off-line HDF or HF and did not enroll any patients receiving OL-HDF. The outcome of interest was QoL of patients with ESRD and we included any RCTs that contained QoL as one of their outcomes regardless of its category as primary or secondary outcome. Any kind of scales assessing QoL were included in this study. We included only RCTs in order to eliminate the disparity of the difference in baseline characteristics of patients which might have affected their QoL.

### Data sources and search strategy

A comprehensive literature search strategy with input from study investigators was designed and carried out by an expert librarian in Mayo Clinic (Patricia J. Erwin) with experience in systematic reviews. The search strategy was performed using MEDLINE, EMBASE, The Cochrane Database of Systematic Reviews, and The Cochrane Central Register of Controlled Trials databases. The timeframe was from the databases inception to February 8, 2018 and no language restriction was employed. The search strategy was highly sensitive and included keywords and several combinations and variations of keywords like: Hemodiafiltration, ESRD, clinical trial, and QoL. Additional references were sought from clinical experts (YU and KT). The complete search strategy can be found on the supplementary material.

### Study selection

**Fig 1:**
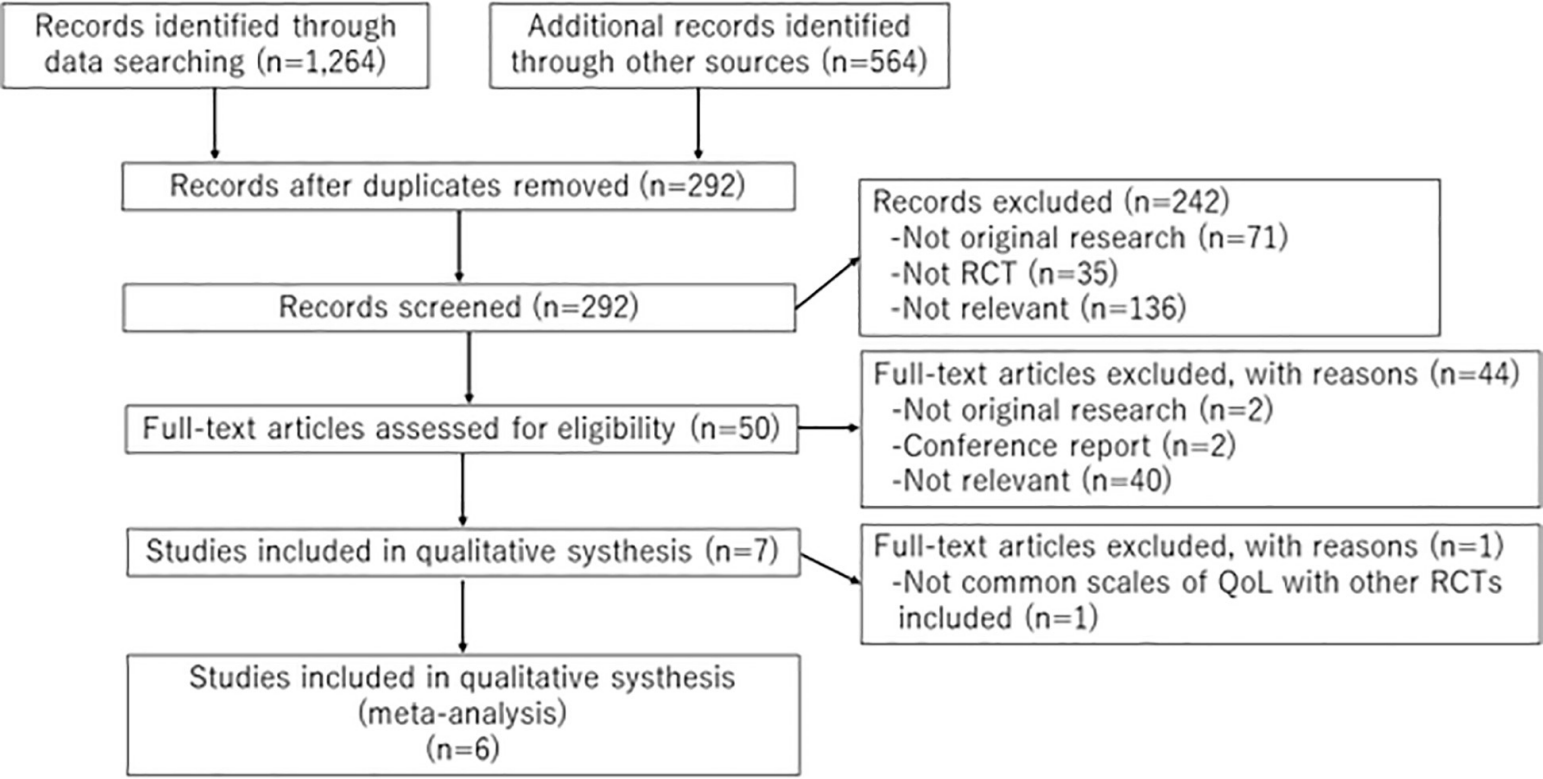
Process of study selection.

The selection process consisted of a title and abstract screening phase and a full- text screening phase (Fig 1). In both phases, each reference was screened by two reviewers in an independently and in duplicate using standardized instructions; one of them was an expert in the content area (TS) and the other reviewer was a non-expert (FJBF). As part of calibration, eligibility criteria were iterated for clarity and consistency. In the title and abstract screening level, both reviewers must have agreed to exclude an article; conflicts were included. Following abstract screening, eligibility of reports was assessed through full-text screening. Disagreements at the full text screening phase were resolved by a consensus between both reviewers. When reviewers couldn’t reach consensus, a third reviewer was consulted (YU or KT).

### Data collection and management

The data from RCTs was extracted using a web-based standardized, and pilot tested data extraction form. Data extraction was done in a duplicated and independent manner by two reviewers (TS and FJBF). We abstracted data on inclusion criteria for each trial, patient demographics, baseline characteristics, sample size, intervention characteristics (parameters related to OL-HDF and HD), follow-up time, scales assessing QoL, before and after scores of QoL, loss to follow-up, and risk of bias indicators. The number of events in each trial was extracted, when available, and attributed to the arm to which patients were randomized.

### Risk of bias assessment

We used the Cochrane risk of bias assessment tool to assess the quality of the primary studies. This tool takes into consideration six domains, (1) random sequence generation, (2) allocation concealment, (3) blinding of participants, (4) incomplete outcome data, (5) selective outcome reporting, and (6) other sources of bias. Two reviewers (TS and FJBF) independently assessed each study´s quality by examining several domains: random sequence generation, allocation concealment, blinding of participants and personnel, blinding of outcome assessment, selective reporting, complete follow up, and other source of bias. Disagreements between the reviewers were resolved by reaching a consensus. When reviewers couldn’t reach consensus, a third reviewer was consulted (YU or RRG). The overall confidence or overall quality of evidence for each outcome was appraised by discussion between the two extractors using the Grading of Recommendations Assessment, Development and Evaluation (GRADE) approach. This approach takes into account the risk of bias of the individual studies, inconsistency in the results, indirectness, imprecision and other considerations to provide a global assessment of the confidence merited by the body of evidence [19].

### Quality of life scales

The summary of QoL scales and assessment is shown in Table 1. Kantartzi et al. used Short Form Health Survey with 36 questions (SF-36) questionnaire [International Quality of Life Assessment Project (IQOLA) SF-36v1 Standard, Greece] to evaluate of QOL [15]. The SF-36 questionnaire is one of the most common methods of evaluating QOL worldwide [20,21]. The 8 domains of the SF-36 can be summarized in 2 scores: the physical component summary score (PCS) and the mental component summary score (MCS); with higher scores indicating a better health status [22]. Final scores have been normalized for the general population with a mean value of 50 and an SD of 10. Mazairac et al., Morena et al. and Smith et al. used Kidney Disease Quality of Life-Short Form (KDQOL-SF) to evaluate of QOL [11,12,14]. KDQOL questionnaire consists of generic (SF-36) and kidney disease-specific (KDQOL) portions with a range 0 to 100. Karkar et al. also used KDQOL-SF version 1.3, but they did no report PCS and MCS [13]. Word et al. used Kidney Disease Questionnaire scored on a seven-point scale, in which 1 is the worst possible score and 7 is the best possible score [16]. Since there were differences in the scales for social activity, fatigue, and emotion, we adjusted these differences by calculating standardized mean difference (SMD). Meanwhile, the scales for PCS and MCS were same in all studies and the mean difference was calculated for PCS and MCS. Schiffl et al. reported only one parameter of QOL (physical symptom of Kidney Disease Questionnaire), therefore we excluded this study by Schiffl et al. from meta-analysis.

**Table 1:** QOL scales and assessment.

Article	QOL scale	SF-PCS, MCS	Other scales (range of score)	Measurement point	Baseline	Follow-up	Δ
Kantartzi 2013	SF-36	◯	8 dimensions (0–100)	3,6,9,12 M		◯	
Karkar 2015	KDOQOL-SF version1.3		16 components (0–100)	2 years		◯	
Mazairac 2013	KDOQOL-SF version1.3	◯	12 domeins (0–100)	Baseline, 1,2,3 years	◯	Possible to calculate	◯
Morena 2017	KDOQOL-SF version1.3	◯	Burden of kidney disease	Baseline, 6,12,18,24 M	Only Graphs	Only Graphs	
Smith 2017	KDOQOL-SF version1.3	◯		Baseline, 4, 8 weeks	◯	◯	
Ward 2000	Kidney disease questionnaire		5 symptoms (1–7)	6,12 M		◯	
Schiffl 2007	Kidney disease questionnaire		Physical symptoms (1–7)	52 weeks		◯	

Description of the QoL scales included in the selected randomized clinical trials and characteristics of the assessment in each study.

◯ = Included, Δ = final vs. baseline difference.

Since most of the studies that evaluated QOL (4/7) included SF-PCS and MCS, we selected these scores for meta-analysis. The rest of scales used in the studies were dissimilar (Table 2). Therefore, we selected “Social activity”, “Fatigue”, and “Emotion” as scales for meta-analysis because they were included at least 3 RCTs. Only 3 of the 6 studies reported QOL score at baseline. Mazairac et al. reported QOL scores at baseline and difference from baseline score, and we estimated QOL scores at follow-up from them. Morena et al. presented only graphs of QOL scores, but after contacting them, they provided us the exact values.

**Table 2:** Other scales of QOL except for PCS and MCS.

Article	Kantartzi 2013	Karkar 2015	Mazairac 2013	Morena 2017	Smith 2017	Ward 2000	Schiffl 2007
Burden of kidney			◯	◯			
Social activity	Social function	◯	Social interaction			Relationship with others	
Physical symptoms						◯	◯
Physical functioning	◯						
Role limitations-physical	◯						
Fatigue	Energy/fatigue	General fatigue				◯	
Emotion	Emotional well-being	General mood				Depression	
Role limitations-emotional	◯						
Frustration						◯	
Effects of kidney disease on daily life			◯				
Work status		◯	◯				
Cognitive function			◯				
Sleep			◯				
Overall health	General health		◯				
Patient satisfaction			◯				
Bodily pain	◯	◯					
Sexual performance		◯					
Appetite		◯					
Taste		◯					
Skin color		◯					
Sport activity		◯					
Sickness		◯					
Cramps		◯					
Itching		◯					
Post-dialysis fatigue		◯					
Joint pain and stiffness		◯					
Body energy		◯					
Dialysis compliance		◯					

Description of other scales (excluding PCS and MCS) included in the selected randomized clinical trials.

◯ = Included

### Summary measures and data synthesis

We estimated the mean differences (MD) and standardized mean differences (SDM) and their 95% confidence interval (CI) and pooled all the studies’ effect size using a random-effects model as described by DerSimonian and Kacker [23]. We chose random-effects model as our main method of analysis because of its conservative summary of estimates and incorporation of between- and within-study variability. To assess heterogeneity of treatment effect among trials, we used the I^2^ statistic; this represents the proportion of heterogeneity of treatment effect across trials that are not attributable to chance or random error. Hence, a value of 50% reflects significant heterogeneity that is due to real differences in study populations, protocols, interventions, or outcomes [24]. The p value threshold for statistical significance was set at .05 for effect sizes. Analyses were conducted using features on RevMan version 5.3 (The Nordic Cochrane Center, Copenhagen, Denmark).

When necessary, we estimated the SD of QoL score at follow-up under the assumption that SD of QoL score at follow-up might be same than that at baseline. Differences in the range of the scales were adjusted by calculating SDM.

Finally, we analyzed the inter-observer agreement in the full text screening phase by calculating Cohen’s kappa coefficient, which resulted in substantial agreement (κ = .737).

## Results

### Characteristics of included studies

**Fig 2:**
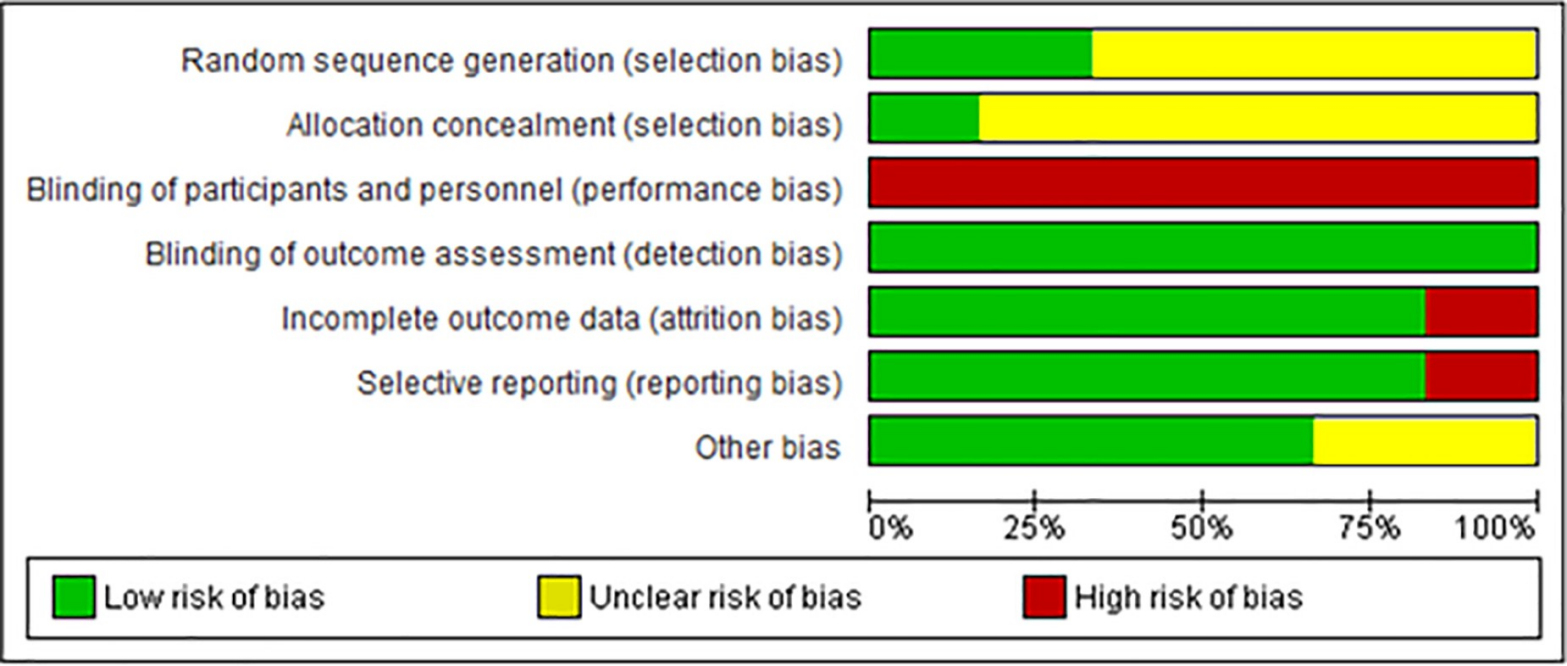
Risk of bias assessment.

A total of 292 studies were identified by our initial electronic search strategy. Of which, 7 RCTs enrolling a total of 1,334 patients met the inclusion criteria. Five studies were carried out in Europe, one study in Saudi Arabia, and 1 study in multiple countries. 2 of them were multi-center trials and the other 5 were single-center. The Convective Transport Study (CONTRAST) was a RCT conducted in 29 dialysis centers in Netherlands, Canada, and Norway to clarify the effect of OL-HDF on all-cause mortality and cardiovascular events [25]. A sub-analysis of CONTRAST focusing on QoL was published by Mazairac et al. in 2013 [14]. 3 of the 7 RCTs had a cross-over design. OL-HDF with post dilution was performed in all the included studies. Convection volume for OL-HDF was over 15 L/session in all the studies. The full-characteristics of the included studies are summarized in Table 3. The primary outcome of the 3 RCTs was QOL, but other 4 RCTs had different primary outcomes. The result of risk of bias assessment is presented in Fig 2. Blinding of participants and personnel was unable to perform in all studies because of the nature of the intervention. Therefore, performance bias was considered high risk for all included studies. Some of the studies included a high rate of lost to follow-up, representing high risk of attrition bias in some RCTs (rates of follow up: Kantartzi et al., 8.3%; Karkar et al., 0%; Mazairac et al., 94.4%; Morena et al., 31.5%; Smith et al., 14%; Ward et al., 13.3%) (S1 Appendix: Summary of findings and confidence in the body of evidence). Our outcomes were QOL scores filled by patients and assessing of these results was objective and less biased. Hence, bias for outcome assessment was considered in low risk. Most of the included articles reported all results in the questionnaire they used. Some articles did not report about random sequence generation and allocation concealment; therefore, they were considered to be unclear. Crossover study design might have been affected by the “carryover effect” in which the effect of one treatment arm was carried to the next one, therefore the category of other bias was considered unclear for crossover design studies.

**Table 3:** Trial description and baseline characteristics.

Author	Year	Place	Study design	N total	N HDF	N HD	Age (mean ± SD) years old	Outcome	Follow-up (years)	Pre or post dilution	Convection volume (L/section)	Comparison
Kantartzi	2013	Greece	Single center cross-over RCT	24	8	8	62.0 ± 13.3	QOL ^†^ Biochemical parameters	1	Post-dilution	15–20	Low-flux HD
Karkar	2015	Saudi Arebia	Single center RCT	72	36	36	54 ± 12	QOL ^†^ Biochemical parameters	2	Post-dilution	19.3 ± 2.1 *	High-flux HD
Mazairac	2013	Netherland Canada Norway	Multicenter RCT	712	356	356	64.1 ± 14.0	QOL ^†^	5	Post-dilution	24	Low-flux HD
Morena	2017	France	Multicenter RCT	381	190	191	76.1 ± 6.7	Intradialytic events ^†^ All-cause of death QOL Biochemical parameters	2	Post-dilution	16.46 ± 2.99 *	High-flux HD
Smith	2017	U.K.	Single center Cross-over RCT	100	50	50	65 ± 14	Recovery time ^†^ Hypotension ^†^ QOL Biochemical parameters	8 weeks	Post-dilution	20.6	High-flux HD
Ward	2000	Germany	Single center RCT	45	24	21	61 ± 3 (OL-HDF) 52 ± 3 (HD)	QOL Biochemical parameters ^†^	1	Post-dilution	21 ± 1 *	High-flux HD
Schiffl	2007	Germany	Single center Cross-over RCT	76	38	38	62 ± 10	QOL Biochemical parameters ^†^ Nutritional status	52 weeks	Post-dilution	4.5 L/hour	High-flux HD

Description of the selected randomized clinical trials and the age of the participants at baseline.

Mean ± SD

^†^ Primary outcome.

### Effect on physical component score (PCS)

**Fig 3:**
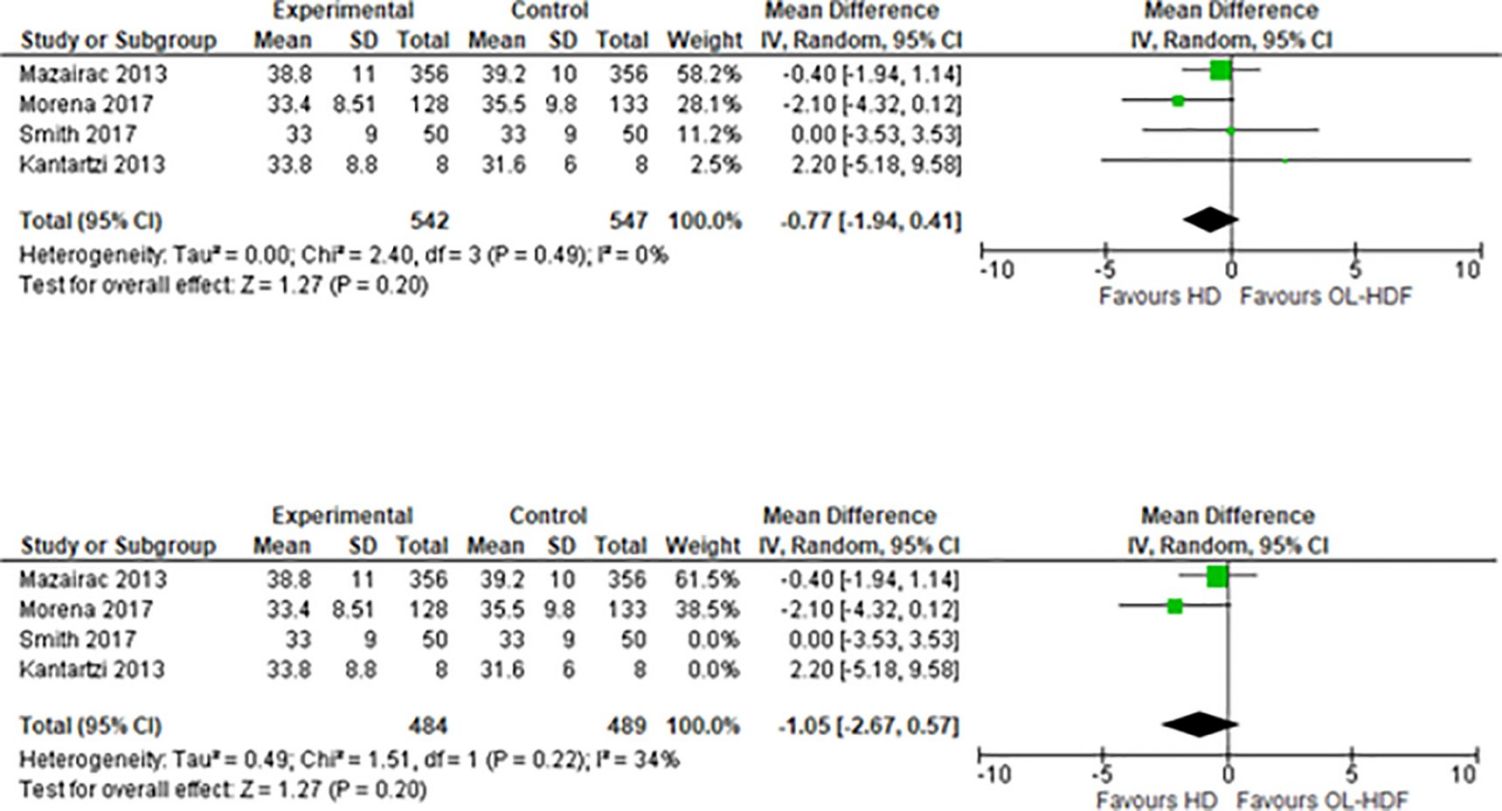
Fig 3–1 Meta-analysis of PCS, Fig 3–2 Sensitivity analysis of PCS.

On the meta-analysis of 4 studies assessing the effect on PCS (1,209 patients), OL-HDF was associated with lower score of PCS compared to HD, but this was not statistically significant: MD -0.77 (95% CI -1.94 to 0.41, p = 0.20) (Fig 3-1). I^2^ = 0%, low heterogeneity.

We then conducted a sensitivity analysis regarding the study design. We excluded the studies that had a cross-over design (Kantartzi 2013 and Smith 2017). This showed that OL-HDF was associated with a lower score of PCS than HD, but this was not statistically significant: MD -1.05 (95% CI -2.67 to 0.57, p = 0.20) (Fig 3-2). I^2^ = 0%, low heterogeneity.

### Effect on mental component score (MCS)

**Fig 4:**
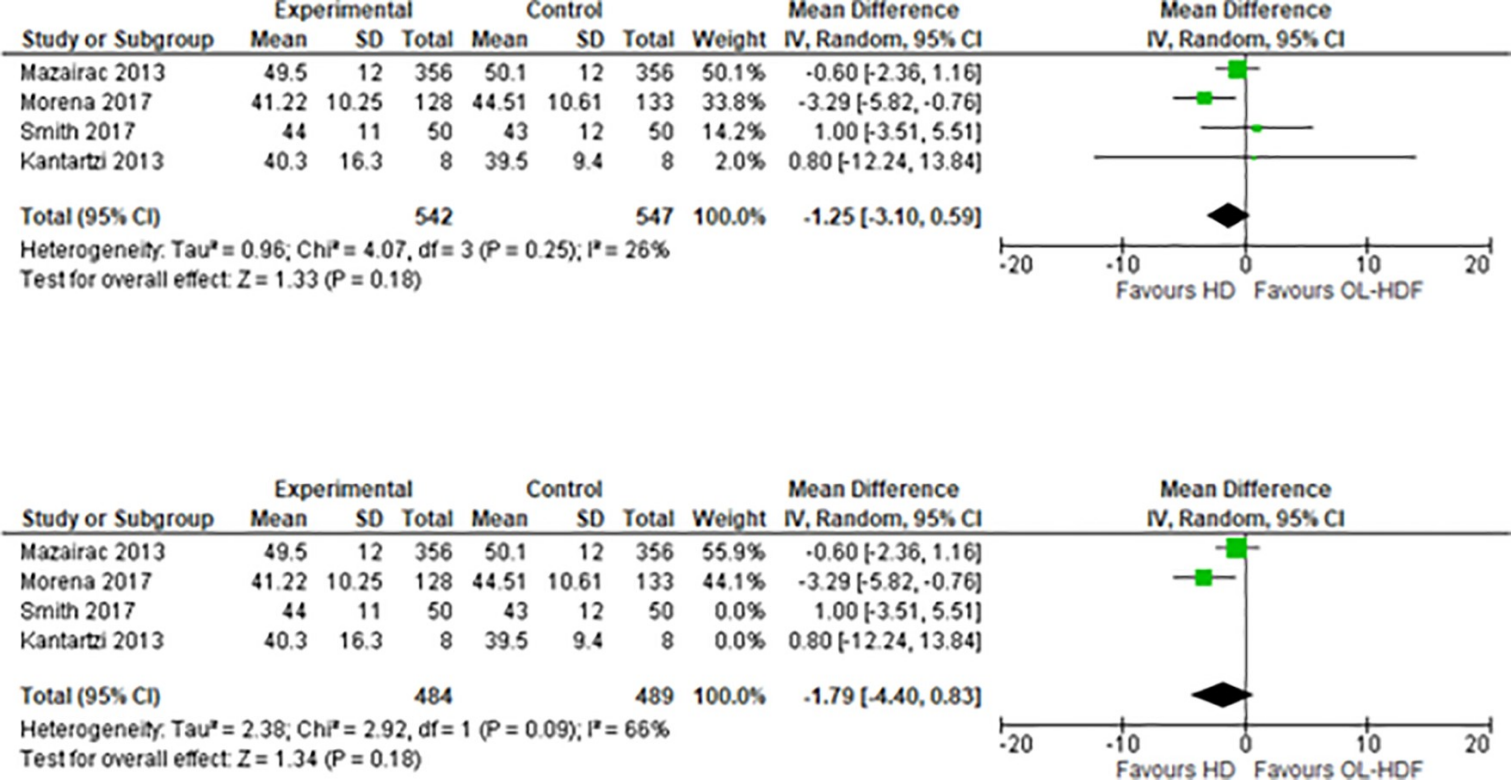
Fig 4–1. Meta-analysis of MCS, Fig 4–2. Sensitivity analysis of MCS.

On the meta-analysis of 4 studies assessing the effect on MCS (1,209 patients), OL-HDF was associated with a lower score in MCS than HD, but this was not statistically significant: MD -1.25 (95% CI -3.10 to 0.59, p = 0.18) (Fig 4-1). I^2^ = 26%, moderate heterogeneity.

We then conducted a sensitivity analysis excluding the studies by Kantartzi 2013 and Smith 2017. This showed that OL-HDF was associated with a lower score of MCS than HD, but this was not statistically significant: MD -1.79 (95% CI -4.40 to 0.83, p = 0.18) (Fig 4-2). I^2^ = 66%, moderate heterogeneity.

### Effect on social activity

**Fig 5:**
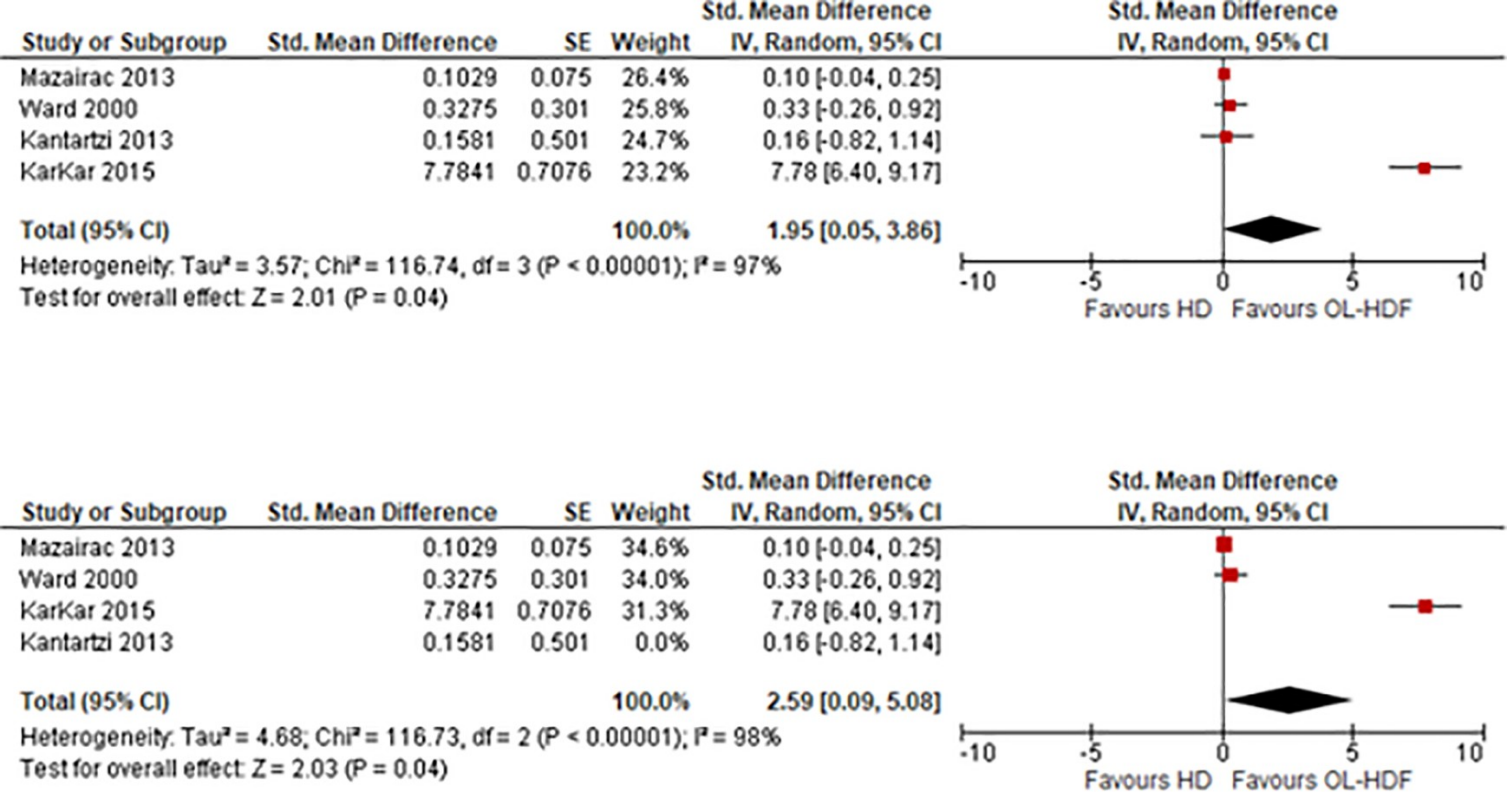
Fig 5–1. Meta-analysis of Social Activity, Fig 5–2. Sensitivity analysis of Social Activity.

3 studies (845 patients) were included for this meta-analysis; where higher scores indicate a better health status. In this meta-analysis, OL-HDF was associated with a statistically significant increase in the score of social activity when compared to HD: SMD 1.95 (95% CI 0.05 to 3.86, p = 0.04) (Fig 5-1). I^2^ = 97%, high heterogeneity.

We then conducted a sensitivity analysis excluding the study by Kantartzi 2013. This showed that OL-HDF was still associated with a statistically significant increase in the score of social activity when compared to HD: SMD 2.59 (95% CI 0.09 to 5.08, p = 0.04) (Fig 5-2). I^2^ = 98%, high heterogeneity.

#### Effect on fatigue

**Fig 6:**
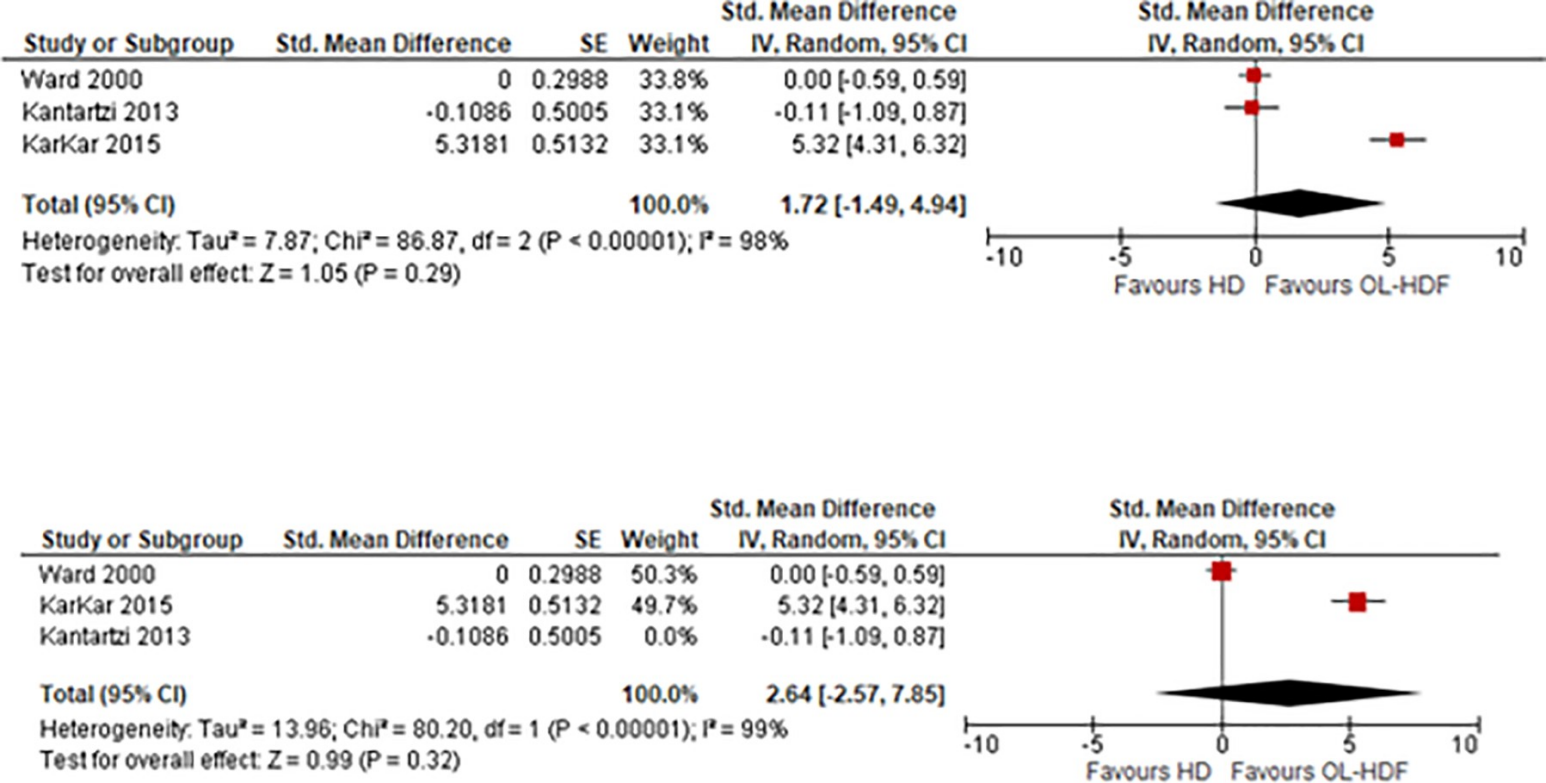
Fig 6–1. Meta-analysis of Fatigue, Fig 6–2. Sensitivity analysis of Fatigue.

Three studies (133 patients) were included for this meta-analysis. Higher scores indicated a better health status in the studies by Kantartzi et al. and Ward et al., but higher scores indicated a worse health status in the study by Karkar, therefore, we inverted the score in the study by Karkar et al. Results showed that, OL-HDF was associated with a non-significant decrease in the score of fatigue when compared to HD: SMD 1.72 (95% CI -1.49 to 4.94, p = 0.29) (Fig 6-1). I^2^ = 98%, high heterogeneity.

We then conducted a sensitivity analysis excluding the study by Kantartzi 2013. This showed that OL-HDF was associated with non-significant decrease in the score of fatigue when compared to HD: SMD 2.64 (95% CI -2.57 to 7.85, p = 0.32) (Fig 6-2). I^2^ = 99%, high heterogeneity.

### Effect on emotion

**Fig 7:**
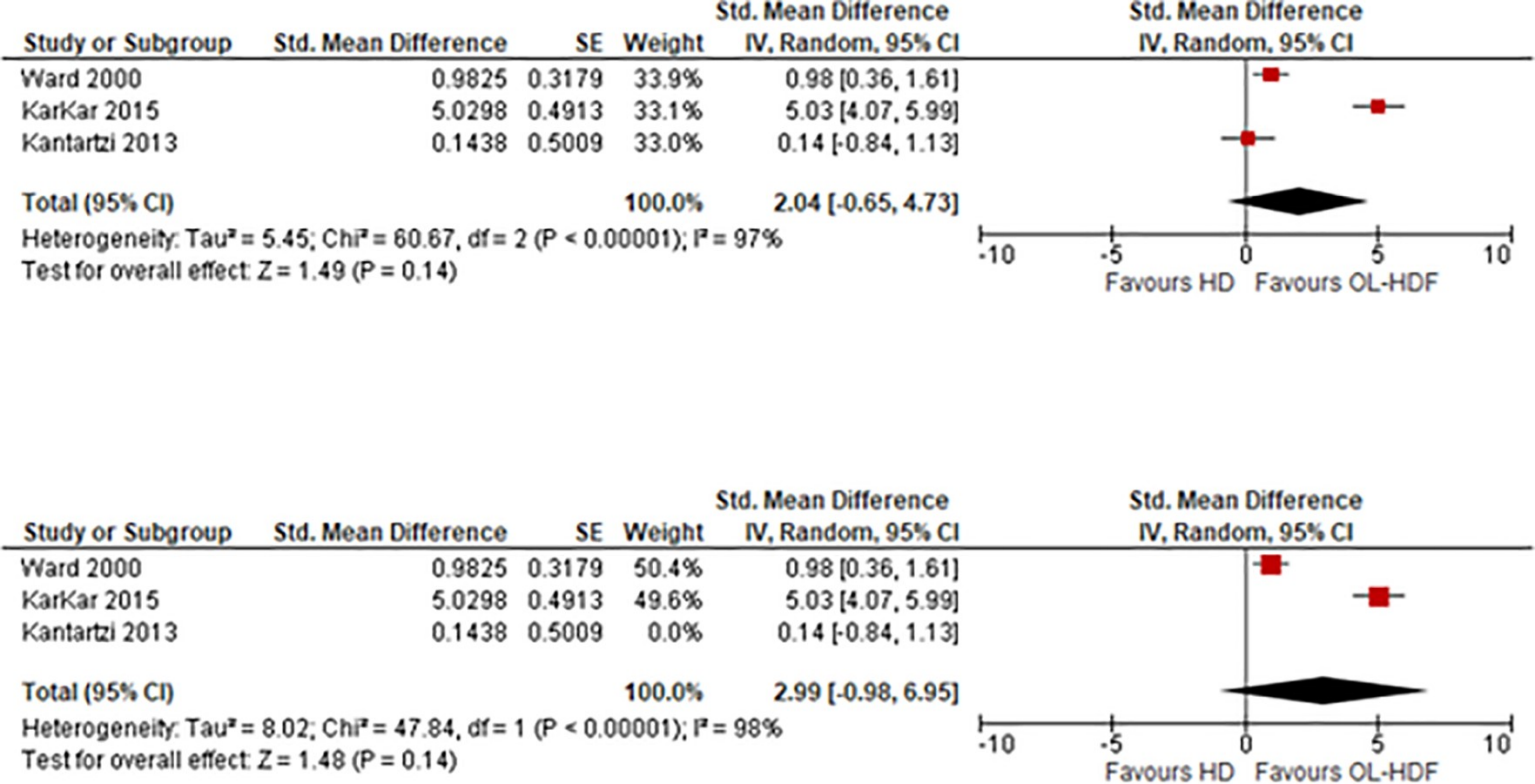
Fig 7–1. Meta-analysis of Emotion, Fig 7–2. Sensitivity analysis of Emotion.

Three studies (133 patients) were included for a meta-analysis with higher scores indicating a better health status. This meta-analysis showed that OL-HDF was associated with a non-significant increase in the score of emotion when compared to HD: SMD 2.04 (95% CI -0.65 to 4.73, p = 0.14) (Fig 7-1). I^2^ = 97%, high heterogeneity.

We then conducted a sensitivity analysis excluding the study by Kantartzi 2013. This showed that OL-HDF was associated with non-significant increase in the score of fatigue when compared to HD: SMD 2.99 (95% CI -0.98 to 6.95, p = 0.14) (Fig 7-2). I^2^ = 98%, high heterogeneity.

### GRADE approach to assess the quality of evidence

Quality of evidence was moderate for the efficacy in Physical Component Score (PCS) and Mental Component Score (MCS) due to risk of bias and nonsignificant effects (S2 Appendix: Loss to Follow Up). In these categories, imprecision (effects were similar among studies) and indirectness (studies reported actual scale scores) was not an issue. Regarding the construct of Social Activity, quality of evidence was assessed as low because of indirectness (studies reported these results in different manners) and risk of bias Although results for this construct were heterogeneous (I^2^ = 95%), there was no substantial variation in the effect estimates across studies. Finally, for the constructs of Fatigue and Emotion, quality of evidence was assessed as very low because of indirectness (different ways of reporting these among studies), imprecision (high heterogeneity and variation in effect estimates across studies), and risk of bias.

## Discussion

### Main findings

This study aimed to assess whether OL-HDF improves QoL of patients compared to HD. We found a significant reduction in the PCS score when comparing OL-HDF versus HD. Nevertheless, this difference was only of 0.81 in the range of 0 to 100. This is a small difference that, despite its statistical significance, we consider that it must not be taken as a real clinical benefit. Regarding MCS, the reduction was also small, only 1.24 points in a range from 0 to 100. We also suggest having some precaution when interpreting these results. Kantartzi et al. reported that convective modalities improved PCS and MCS as well as bodily pain when compared to HD. Ward et al. did not report PCS and MCS, but they reported that, using the Kidney Disease Questionnaire, OL-HDF resulted in a significant improvement of physical symptoms when compared to HD. Mazairac et al. and Morena et al. reported that OL-HDF was related to a lower score in the PCS and MCS when compared to HD, though they were not significant. Taking this, and our meta-analytic results into account, we consider that the actual body of evidence fails to demonstrate a real improvement in PCS and MCS scores when comparing OL-HDF versus regular HD.

Regarding the other scales of QoL (Social activity, Fatigue, Emotion), all of the standardized mean differences between OL-HDF and HD were small except for those in the study by Karkar et al [13]. The results of the study by Karkar et al. resulted in incremental benefit of 7.8 SMD, 5.3 SMD, and 5.0 SMD, in the scores of Social activity, Fatigue, and Emotion, respectively when comparing OL-HDF with HD. This large difference in results between the study by Karkar et al. and other studies might have been the reason for high heterogeneity in these scales of QoL. This study, used post-dilution OL-HDF with an average substitution fluid of 19.3 ± 2.1 liter per section for a total follow-up time of 24 months. These parameters are similar with those in the rest of the included studies in this analysis (Kantartzi et al. and Ward et al.) However, the positive results of Karkar et al. might have been influenced by younger age of samples (mean age of patients: 54.0 vs 62.0 vs 61 years old) and a different geographic region of the primary site (Saudi Arabia vs Europe). [15,16]. Another limitation when analyzing the study by Karkar et al. was that we didn’t know if the baseline score for QoL was same for both groups because they did not report the baseline score. Nevertheless, their results suggest that there might be an improvement in QoL, especially for younger patients, when comparing OL-HDF versus regular HD.

### Implications for clinical practice and research

QoL of patients is an important clinical outcome as well as mortality, and it’s also a predictor for morbidity and mortality [26]. Previous studies have suggested a possibility of improvement in QoL of patients even in a short period by reporting that convective therapies can reduce the incidence of intradialytic hypotension and that OL-HDF is associated with greater hemodynamic stability[27]. Surprisingly, our results suggest that there isn’t a real benefit provided by OL-HDF on QoL when compared with regular HD within a time frame of five years of follow-up. Better removal of middle molecules, β2 microglobulin, and phosphorus could also improve clinical symptoms of patients with ESRD such as itching. Comparison of HD and hemodiafiltration with respect to QoL has produced contradictory results so far. A relatively large observational study by Canaud et al. reported that there was no difference across the dialysis modalities when analyzing QoL of patients with ESRD. Meanwhile, a cross-sectional study using SF-36 by Knezwic et al. reported that QoL in patients on OL-HDF was significantly better than that in patients on HD with respect to most scales of SF-36 [28]. Nevertheless, there is a difference in the mean age of the patients included in these studies, 63.5 and 56 years old in the study of Canaud et al. and Knezevic et al., respectively. Therefore, this imbalance in age may have influenced the differences in their results. The influence of this confounding factor (age) can be supported also by the results of the study of Karkar et al. Therefore, we should reconsider the administration of OL-HDF as a dialytic modality for treatment in elderly patients because an improvement in QoL might not be achieved in a short period. On the other hand, OL-HDF might be an effective option for improving QoL in younger patients. Future RCTs should be done in larger number of young patients to clarify the effectiveness of OL-HDF on QoL of patients with ESRD.

OL-HDF is thought to be associated better removal of both small and middle sized molecules including β2 microglobulin [25,29]. Therefore, this modality could lead to an improvement in the long-term complications of dialysis such as secondary amyloidosis. Nevertheless, prospective studies with longer follow up periods would be necessary to evaluate this possible long-term effectiveness of OL-HDF on QoL. Convection volume can be another important factor to determine the effectiveness of this modality over conventional HD. However, average convection volume in all the included studies was more than 15 L/section. Consequently, we couldn’t assess the effect of convection volume on QoL on this study.

### Strengths and limitations

One strength of this study was that we included only RCTs. In addition, we focused on OL-HDF and excluded other convective therapies (off-line HDF or HF) to reduce the variability in the effectiveness across convective therapies. In addition, the treatment-parameters used with OL-HDF were relatively similar in all included studies. For example, all studies used post-dilution modality and all of them had an average convection volume greater than 15L. There are also some limitations in this study. First, as a comparison of OL-HDF, some studies included patients on low-flux HD and others included patients on high-flux HD. The characteristics of enrolled patients were also variable among each study. Morena et al. targeted only elderly patients over 65, and such differences might have influenced the results of this study. Also, regarding the assessment of QoL, the follow-up periods were various and ranged from 8 weeks to 5 years. This difference in follow up time might have affected the results of our meta-analysis as well. The evaluation of QoL was assessed by patients themselves; therefore, scores could be quite subjective. All studies were unblinded and QoL was evaluated through questionnaires; therefore, the risk of bias was high. Some papers did not provide the baseline data, therefore we estimated some missing scores by calculating them, which might have introduced some measurement bias. In addition, 2 of the 6 RCTs were crossover design. Since most of the studies included in this meta-analysis were conducted in Europe, we can’t truly generalize these results to the other part of the world. Also, the number of included studies was small and this made it difficult to evaluate the risk of publication bias. At last, the number of studies included in this meta-analysis is limited, therefore accumulation of studies would be necessary to make stronger conclusions.

### Conclusion

The body of evidence shows that OL-HDF does not improve QoL of patients when compared to HD within a 5-year follow-up period. OL-HDF, yet, may be an effective option to improve QoL in non-elderly patients. More and better quality evidence, however, is needed to have a greater confidence and certainty in the estimates.

## Supporting information

S1 Appendix Summary of findings and confidence in the body of evidence. (DOCX)

S2 Appendix Loss to follow up. (DOCX)

S3 Appendix Search strategy. (DOCX)

S4 Appendix List of excluded studies. (DOCX)

S5 Appendix PRISMA 2009 checklist. (DOC)

S6 Appendix PRISMA 2009 flow diagram. (DOC)
