# Supplementary material for: Effect of online hemodiafiltration compared with hemodialysis on quality of life in patients with ESRD: A systematic review and meta-analysis of randomized trials
Source: PLoS One. 2018 Oct 18;13(10):e0205037. doi: 10.1371/journal.pone.0205037 (PMC6193628; doi:10.1371/journal.pone.0205037)
Supplement: S1 Appendix — (DOCX) [file pone.0205037.s001.docx]

**(S1 Appendix) Loss to Follow Up**

| **Study** | **n/N** | **Percentage of loss of follow up** |
| --- | --- | --- |
| Kantartzi, 2013 | 22/24 | 8.3% |
| Karkar, 2015 | 72/72 | 0% |
| Mazairac, 2013 | 40/712 | 94.4% |
| Morena, 2017 | 261/381 | 31.5% |
| Smith, 2017 | 86/100 | 14% |
| Ward, 2000 | 39/45 | 13.3% |
