# Supplementary material for: Effect of online hemodiafiltration compared with hemodialysis on quality of life in patients with ESRD: A systematic review and meta-analysis of randomized trials
Source: PLoS One. 2018 Oct 18;13(10):e0205037. doi: 10.1371/journal.pone.0205037 (PMC6193628; doi:10.1371/journal.pone.0205037)
Supplement: S2 Appendix — (DOCX) [file pone.0205037.s002.docx]

**(S2 Appendix) Summary of findings and confidence in the body of evidence**

| **Efficacy Outcomes by Scales** | **Effect Size (95% Ci)** | **No of Participants (Total Studies)** | **Quality of Evidence (Domains of Concern)** |
| --- | --- | --- | --- |
| Physical Component Score | MD: -0.77 (95% CI, -1.94, 0.41) | 1,209 (4) | Moderate (Risk of Bias) |
| Mental Component Score | MD: -1.25 (95% CI, -3.10, 0.59) | 1,209 (4) | Moderate (Risk of Bias) |
| **Efficacy Outcomes by Construct** |  |  |  |
| Social Activity | SMD: 1.95 (95% CI, 0.05, 3.86) | 845 (3) | Low (Indirectness, Risk of Bias) |
| Fatigue | SMD: 1.72 (95% CI, -1.49, 4.94) | 133 (3) | Very Low (Imprecision, Indirectness, Risk of Bias) |
| Emotion | SMD: 2.04 (95% CI, -0.65, 4.73) | 133 (3) | Very Low (Imprecision, Indirectness, Risk of Bias) |
