# Supplementary material for: Effect of online hemodiafiltration compared with hemodialysis on quality of life in patients with ESRD: A systematic review and meta-analysis of randomized trials
Source: PLoS One. 2018 Oct 18;13(10):e0205037. doi: 10.1371/journal.pone.0205037 (PMC6193628; doi:10.1371/journal.pone.0205037)
Supplement: S3 Appendix — (DOCX) [file pone.0205037.s003.docx]

**(S3 Appendix) Search strategy**

**Ovid MEDLINE(R) Epub Ahead of Print, In-Process & Other Non-Indexed Citations, Ovid MEDLINE(R) Daily and Ovid MEDLINE(R) <1946 to Present>**

| Search history sorted by search number ascending | | | |  |  |  |
| --- | --- | --- | --- | --- | --- | --- |
| **#** | **Searches** | **Results** | **Type** |  |  |  |
|  | | | | | | |
| 1 | Hemodiafiltration/ | 2331 | Advanced |  |  |  |
| 2 | (haemodiafiltration or haemo-diafiltration or hemodiafiltration or hemo diafiltration).mp. [mp=title, abstract, original title, name of substance word, subject heading word, keyword heading word, protocol supplementary concept word, rare disease supplementary concept word, unique identifier, synonyms] | 3623 | Advanced |  |  |  |
| 3 | 1 or 2 | 3623 | Advanced |  |  |  |
| 4 | (ol adj hdf).mp. [mp=title, abstract, original title, name of substance word, subject heading word, keyword heading word, protocol supplementary concept word, rare disease supplementary concept word, unique identifier, synonyms] | 116 | Advanced |  |  |  |
| 5 | (online or ("on" adj line)).mp. | 112684 | Advanced |  |  |  |
| 6 | 3 and (4 or 5) | 670 | Advanced |  |  |  |
| 7 | ("end stage" adj2 (renal or kidney)).mp. [mp=title, abstract, original title, name of substance word, subject heading word, keyword heading word, protocol supplementary concept word, rare disease supplementary concept word, unique identifier, synonyms] | 40015 | Advanced |  |  |  |
| 8 | (esrd or eskd).mp. [mp=title, abstract, original title, name of substance word, subject heading word, keyword heading word, protocol supplementary concept word, rare disease supplementary concept word, unique identifier, synonyms] | 16058 | Advanced |  |  |  |
| 9 | exp Renal Insufficiency/ | 167255 | Advanced |  |  |  |
| 10 | ((renal or kidney) adj2 (failure* or chronic*)).mp. [mp=title, abstract, original title, name of substance word, subject heading word, keyword heading word, protocol supplementary concept word, rare disease supplementary concept word, unique identifier, synonyms] | 209008 | Advanced |  |  |  |
| 11 | or/7-10 | 248790 | Advanced |  |  |  |
| 12 | 6 and 11 | 357 | Advanced |  |  |  |
| 13 | 6 and (outcome* or followup* or follow-up* or recur* or surviv* or compar* or death* or mortality or events or complicat* or quality of life or QOL or QoL).mp. [mp=title, abstract, original title, name of substance word, subject heading word, keyword heading word, protocol supplementary concept word, rare disease supplementary concept word, unique identifier, synonyms] | 498 | Advanced |  |  |  |
| 14 | limit 12 to (clinical study or clinical trial, all or clinical trial, phase i or clinical trial, phase ii or clinical trial, phase iii or clinical trial, phase iv or clinical trial or comparative study or controlled clinical trial or evaluation studies or meta analysis or multicenter study or observational study or pragmatic clinical trial or randomized controlled trial or systematic reviews) | 173 | Advanced |  |  |  |
| 15 | 12 and (observational* or study or studies or "case series" or "case control*" or random* or trial* or prospective* or retrospective* or compar* or follow*).mp. [mp=title, abstract, original title, name of substance word, subject heading word, keyword heading word, protocol supplementary concept word, rare disease supplementary concept word, unique identifier, synonyms] | 289 | Advanced |  |  |  |
| 16 | 13 or 14 or 15 | 524 | Advanced |  |  |  |
| 17 | remove duplicates from 16 | 480 |  |  |  |  |

**EBM Reviews - Cochrane Central Register of Controlled Trials**

| Search history sorted by search number ascending | | | |  |  |  |
| --- | --- | --- | --- | --- | --- | --- |
| **#** | **Searches** | **Results** | **Type** |  |  |  |
|  | | | | | | |
| 1 | Hemodiafiltration/ | 209 | Advanced |  |  |  |
| 2 | (haemodiafiltration or haemo-diafiltration or hemodiafiltration or hemo diafiltration).mp. [mp=title, original title, abstract, mesh headings, heading words, keyword] | 472 | Advanced |  |  |  |
| 3 | 1 or 2 | 472 | Advanced |  |  |  |
| 4 | (ol adj hdf).mp. [mp=title, original title, abstract, mesh headings, heading words, keyword] | 37 | Advanced |  |  |  |
| 5 | (online or ("on" adj line)).mp. | 28272 | Advanced |  |  |  |
| 6 | 3 and (4 or 5) | 194 | Advanced |  |  |  |
| 7 | ("end stage" adj2 (renal or kidney)).mp. [mp=title, original title, abstract, mesh headings, heading words, keyword] | 2489 | Advanced |  |  |  |
| 8 | (esrd or eskd).mp. [mp=title, original title, abstract, mesh headings, heading words, keyword] | 1082 | Advanced |  |  |  |
| 9 | exp Renal Insufficiency/ | 5520 | Advanced |  |  |  |
| 10 | ((renal or kidney) adj2 (failure* or chronic*)).mp. [mp=title, original title, abstract, mesh headings, heading words, keyword] | 13309 | Advanced |  |  |  |
| 11 | or/7-10 | 14877 | Advanced |  |  |  |
| 12 | 6 and 11 | 104 | Advanced |  |  |  |
| 13 | 6 and (outcome* or followup* or follow-up* or recur* or surviv* or compar* or death* or mortality or events or complicat* or quality of life or QOL or QoL).mp. [mp=title, original title, abstract, mesh headings, heading words, keyword] | 163 | Advanced |  |  |  |
| 14 | 12 or 13 | 173 |  |  |  |  |
|  |  |  |  |  |  |  |

**Embase <1988 to 2018 Week 03>**

| \| Search history sorted by search number ascending \| \| \| \| \| --- \| --- \| --- \| --- \| \| **#** \| **Searches** \| **Results** \| **Type** \| \|  \| \| \| \| \| \| \| \| 1 \| Hemodiafiltration/ \| 2439 \| Advanced \|  \|  \|  \| \| 2 \| (haemodiafiltration or haemo-diafiltration or hemodiafiltration or hemo diafiltration).mp. [mp=title, abstract, heading word, drug trade name, original title, device manufacturer, drug manufacturer, device trade name, keyword, floating subheading word] \| 5097 \| Advanced \|  \|  \|  \| \| 3 \| 1 or 2 \| 5097 \| Advanced \|  \|  \|  \| \| 4 \| (ol adj hdf).mp. [mp=title, abstract, heading word, drug trade name, original title, device manufacturer, drug manufacturer, device trade name, keyword, floating subheading word] \| 182 \| Advanced \|  \|  \|  \| \| 5 \| (online or ("on" adj line)).mp. \| 143521 \| Advanced \|  \|  \|  \| \| 6 \| 3 and (4 or 5) \| 913 \| Advanced \|  \|  \|  \| \| 7 \| ("end stage" adj2 (renal or kidney)).mp. [mp=title, abstract, heading word, drug trade name, original title, device manufacturer, drug manufacturer, device trade name, keyword, floating subheading word] \| 55572 \| Advanced \|  \|  \|  \| \| 8 \| (esrd or eskd).mp. [mp=title, abstract, heading word, drug trade name, original title, device manufacturer, drug manufacturer, device trade name, keyword, floating subheading word] \| 22651 \| Advanced \|  \|  \|  \| \| 9 \| exp Renal Insufficiency/ \| 254915 \| Advanced \|  \|  \|  \| \| 10 \| ((renal or kidney) adj2 (failure* or chronic*)).mp. [mp=title, abstract, heading word, drug trade name, original title, device manufacturer, drug manufacturer, device trade name, keyword, floating subheading word] \| 295044 \| Advanced \|  \|  \|  \| \| 11 \| or/7-10 \| 337357 \| Advanced \|  \|  \|  \| \| 12 \| 6 and 11 \| 420 \| Advanced \|  \|  \|  \| \| 13 \| 6 and (outcome* or followup* or follow-up* or recur* or surviv* or compar* or death* or mortality or events or complicat* or quality of life or QOL or QoL).mp. [mp=title, abstract, heading word, drug trade name, original title, device manufacturer, drug manufacturer, device trade name, keyword, floating subheading word] \| 707 \| Advanced \|  \|  \|  \| \| 14 \| 12 or 13 \| 778 \| Advanced \|  \|  \|  \| \| 15 \| exp case control study/ or exp case study/ or exp clinical trial/ or exp longitudinal study/ or exp major clinical study/ or exp prospective study/ or exp retrospective study/ \| 3936053 \| Advanced \|  \|  \|  \| \| 16 \| exp comparative study/ or exp controlled study/ or exp observational study/ \| 6474991 \| Advanced \|  \|  \|  \| \| 17 \| 14 and (15 or 16) \| 413 \| Advanced \|  \|  \|  \| \| 18 \| 14 and (study* or studies).mp. [mp=title, abstract, heading word, drug trade name, original title, device manufacturer, drug manufacturer, device trade name, keyword, floating subheading word] \| 611 \| Advanced \|  \|  \|  \| \| 19 \| 17 or 18 \| 628 \| Advanced \|  \|  \|  \| \| 20 \| remove duplicates from 19 \| 611 \|  \|  \|  \|  \| |
| --- | --- | --- | --- | --- | --- | --- | --- | --- | --- | --- | --- | --- | --- | --- | --- | --- | --- | --- | --- | --- | --- | --- | --- | --- | --- | --- | --- | --- | --- | --- | --- | --- | --- | --- | --- | --- | --- | --- | --- | --- | --- | --- | --- | --- | --- | --- | --- | --- | --- | --- | --- | --- | --- | --- | --- | --- | --- | --- | --- | --- | --- | --- | --- | --- | --- | --- | --- | --- | --- | --- | --- | --- | --- | --- | --- | --- | --- | --- | --- | --- | --- | --- | --- | --- | --- | --- | --- | --- | --- | --- | --- | --- | --- | --- | --- | --- | --- | --- | --- | --- | --- | --- | --- | --- | --- | --- | --- | --- | --- | --- | --- | --- | --- | --- | --- | --- | --- | --- | --- | --- | --- | --- | --- | --- | --- | --- | --- | --- | --- | --- | --- | --- | --- | --- | --- | --- | --- | --- | --- | --- | --- | --- | --- | --- | --- | --- | --- | --- | --- | --- | --- | --- | --- | --- | --- |

Web of Science

**TOPIC:** ((haemodiafiltration OR "haemo-diafiltration" OR hemodiafiltration OR "hemo diafiltration" OR HDF) NEAR ("on-line" OR online OR ol)) *AND* **TOPIC:** (outcome* OR death* OR surviv* OR clearance OR levels OR follow* OR event* OR complicat* OR hospitali*) **564**
