# Supplementary material for: Effect of online hemodiafiltration compared with hemodialysis on quality of life in patients with ESRD: A systematic review and meta-analysis of randomized trials
Source: PLoS One. 2018 Oct 18;13(10):e0205037. doi: 10.1371/journal.pone.0205037 (PMC6193628; doi:10.1371/journal.pone.0205037)
Supplement: S4 Appendix — (DOCX) [file pone.0205037.s004.docx]

**(S4 Appendix) List of excluded studies**

1. Ahrenholz P, Taborsky P, Bohling M, Rawer P, Ibrahim N, Gajdos M, et al. Determination of dialysis dose: A clinical comparison of methods. Blood Purification. 2011; 32: 271-277. http://dx.doi.org/10.1159/000330340. PMID: 51585268.

2. Ahrenholz P, Winkler RE, Ramlow W, Tiess M, Muller W. On-line hemodiafiltration with pre- and postdilution: a comparison of efficacy. International Journal of Artificial Organs. 1997; 20: 81-90. PMID: 9093885.

3. Ahrenholz P, Winkler RE, Ramlow W, Tiess M, Thews O. On-line hemodiafiltration with pre- and postdilution: impact on the acid-base status. International Journal of Artificial Organs. 1998; 21: 321-327. PMID: 9714025.

4. Ahrenholz PG, Winkler RE, Michelsen A, Lang DA, Bowry SK. Dialysis membrane-dependent removal of middle molecules during hemodiafiltration: the beta2-microglobulin/albumin relationship. Clinical Nephrology. 2004; 62: 21-28. PMID: 15267009.

5. Akizawa T, Koiwa F. Clinical Expectation of Online Hemodiafiltration: A Japanese Perspective. Blood Purification. 2015; 40 Suppl 1: 12-16. https://dx.doi.org/10.1159/000437405. PMID: 26344508.

6. Alamartine E, de Filippis JP, Toulon J, Berthoux F. On-line continuous venovenous hemodiafiltration: a technique for the control of ultrafiltration and convection during continuous renal replacement therapy. Renal Failure. 1994; 16: 707-714. PMID: 7899582.

7. Albalate Ramon M, Perez Garcia R, de Sequera Ortiz P, Alcazar Arroyo R, Corchete Prats E, Puerta Carretero M, et al. Clinical application of Ultracontrol: infusion volume and use with different dialyzers. Nefrologia. 2011; 31: 683-689. https://dx.doi.org/10.3265/Nefrologia.pre2011.Sep.11122. PMID: 22130284.

8. Altieri P, Sau G, Menneas A, Cabiddu G, Michittu MB, Mereu MC. [Are convective treatments equivalent to the traditional ones? The Hemo Study and beyond (review)]. Giornale Italiano di Nefrologia. 2004; 21: 245-253. PMID: 15285003.

9. Altieri P, Sorba G, Bolasco P, Ledebo I, Ganadu M, Ferrara R, et al. Comparison between hemofiltration and hemodiafiltration in a long-term prospective cross-over study. Journal of Nephrology. 2004; 17: 414-422. PMID: 15365963.

10. Amato M, Brendolan A, Campolo G, Petras D, Bonello M, Crepaldi C, et al. Sequential hemofiltration-hemodiafiltration technique: all in one? Contributions to Nephrology. 2005; 149: 115-120. https://dx.doi.org/10.1159/000085471. PMID: 15876835.

11. Andrulli S, Altieri P, Sau G, Bolasco P, Pedrini LA, Basile C, et al. Predictors of haemoglobin levels and resistance to erythropoiesis-stimulating agents in patients treated with low flux hemodialysis, hemofiltration and hemodiafiltration: Results of a multicentre, randomized and controlled trial. Nephrology Dialysis Transplantation. 2012; 2): ii215. http://dx.doi.org/10.1093/ndt/gfs224. PMID: 70765926.

12. Arese M, Cristol JP, Bosc JY, Bussolino F, Wratten ML, Tetta C, et al. Removal of constitutive and inducible nitric oxide synthase-active compounds in a modified hemodiafiltration with on-line production of substitution fluid: the contribution of convection and diffusion. International Journal of Artificial Organs. 1996; 19: 704-711. PMID: 9029246.

13. Argyropoulos C, Roumelioti ME, Sattar A, Kellum JA, Weissfeld L, Unruh ML. Dialyzer Reuse and Outcomes of High Flux Dialysis. Plos One. 2015; 10: e0129575. 10.1371/journal.pone.0129575. PMID: WOS:000356349000063.

14. Ariza F, Merino A, Carracedo J, Alvarez de Lara MA, Crespo R, Ramirez R, et al. Post-dilution high convective transport improves microinflammation and endothelial dysfunction independently of the technique. Blood Purification. 2013; 35: 270-278. https://dx.doi.org/10.1159/000350611. PMID: 23689471.

15. Arnold R, Pussell BA, Pianta TJ, Grinius V, Lin CS, Kiernan MC, et al. Effects of hemodiafiltration and high flux hemodialysis on nerve excitability in end-stage kidney disease. PLoS ONE [Electronic Resource]. 2013; 8: e59055. https://dx.doi.org/10.1371/journal.pone.0059055. PMID: 23536855.

16. Arrigo G, Beltrame A, Colasanti G. Adequacy in hemodialysis: new concepts derived from online urea monitoring. Kidney International - Supplement. 2000; 76: S41-46. PMID: 10936798.

17. Aydemir B, Gunes B, Mirza C, Gozkonan A, Papila R, Unal E, et al. Managing anemia via adjusting restitution volumes is an effective, safe and economical way to control anemia. Nephrology Dialysis Transplantation. 2017; 32 (Supplement 3): iii341-iii342. http://dx.doi.org/10.1093/ndt/gfx153. PMID: 617290247.

18. Barta K, Czifra A, Kun C, Pall A, Kulcsar J, Paragh G, et al. Hemodiafiltration beneficially affects QT interval duration and dispersion compared to hemodialysis. Clinical and Experimental Nephrology. 2014; 18: 952-959. 10.1007/s10157-014-0950-9. PMID: WOS:000346636600018.

19. Basile C, Davenport A, Blankestijn PJ. Why choose high volume online post-dilution hemodiafiltration? Journal of Nephrology. 2017; 30: 181-186. https://dx.doi.org/10.1007/s40620-016-0343-0. PMID: 27586123.

20. Bataille P, Celine P, Raymond A, Francois G, Herve L, Michel D, et al. Effects on nutritional markers of haemodiafiltration with regeneration of ultrafiltrate (HFR) versus high flux dialysis: A cross-over randomized trial multicentre trial. Nephrology Dialysis Transplantation. 2012; 2): ii199. http://dx.doi.org/10.1093/ndt/gfs224. PMID: 70765881.

21. Bellien J, Freguin-Bouilland C, Joannides R, Hanoy M, Remy-Jouet I, Monteil C, et al. High-efficiency on-line haemodiafiltration improves conduit artery endothelial function compared with high-flux haemodialysis in end-stage renal disease patients. Nephrology Dialysis Transplantation. 2014; 29: 414-422. https://dx.doi.org/10.1093/ndt/gft448. PMID: 24235073.

22. Bolasco P, Altieri P, Andrulli S, Basile C, Di Filippo S, Feriani M, et al. Convection versus diffusion in dialysis: an Italian prospective multicentre study. Nephrology Dialysis Transplantation. 2003; 18 Suppl 7: vii50-54; discussion vii59-62. PMID: 12953031.

23. Bolasco PG. [Pro convection: are convective therapies like hemodiafiltration and hemofiltration the future of extracorporeal blood purification?]. Giornale Italiano di Nefrologia. 2008; 25: 389-395. PMID: 18663685.

24. Bonforte G, Grillo P, Zerbi S, Surian M. Improvement of anemia in hemodialysis patients treated by hemodiafiltration with high-volume on-line-prepared substitution fluid. Blood Purification. 2002; 20: 357-363. https://dx.doi.org/63104. PMID: 12169845.

25. Bonomini M, Ballone E, Di Stante S, Bucciarelli T, Dottori S, Arduini A, et al. Removal of uraemic plasma factor(s) using different dialysis modalities reduces phosphatidylserine exposure in red blood cells. Nephrology Dialysis Transplantation. 2004; 19: 68-74. PMID: 14671041.

26. Bosc JY, LeBlanc M, Garred LJ, Marc JM, Foret M, Babinet F, et al. Direct determination of blood recirculation rate in hemodialysis by a conductivity method. ASAIO journal (American Society for Artificial Internal Organs :. 1998; 1992) 44: 68-73. PMID: CN-00147527 UPDATE.

27. Bosch-Panadero E, Fontao SM, Sanchez-Espinosa D, Camarero V, Gomez MVP, Priego AR, et al. Diferencies in bisphenol a (BPA) serum levels in online hemodiafiltration hemodialysis (HDF) patients with two different membranes. Nephrology Dialysis Transplantation. 2017; 32 (Supplement 3): iii293. http://dx.doi.org/10.1093/ndt/gfx150. PMID: 617291097.

28. Bots ML, Den Hoedt C, Pc Grooteman M, Van Der Weerd NC, Mazairac AHA, Levesque R, et al. The effect of online hemodiafiltration on systemic inflammation in a randomized controlled trial: Results from the convective transport study (contrast). Nephrology Dialysis Transplantation. 2012; 27: ii206. PMID: CN-01005265 UPDATE.

29. Bourguignon C, Chenine L, Bargnoux AS, Leray-Moragues H, Canaud B, Cristol JP, et al. Hemodiafiltration improves free light chain removal and normalizes kappa/lambda ratio in hemodialysis patients. Journal of Nephrology. 2016; 29: 251-257. https://dx.doi.org/10.1007/s40620-015-0207-z. PMID: 26022721.

30. Bowry SK, Canaud B. Clinical benefits of post-dilution online haemdiafiltration. Blood Purification. 2013; 34 (3-4): 266. http://dx.doi.org/10.1159/000345376. PMID: 71012309.

31. Bowry SK, Canaud B. Achieving high convective volumes in on-line hemodiafiltration. Blood Purification. 2013; 35 Suppl 1: 23-28. https://dx.doi.org/10.1159/000346379. PMID: 23466374.

32. Buturovic-Ponikvar J, Gubensek J, Ponikvar R. Citrate anticoagulation for postdilutional online hemodiafiltration with calcium-containing dialysate and infusate: significant clotting in the venous bubble trap. International Journal of Artificial Organs. 2008; 31: 323-328. PMID: 18432588.

33. Calo LA, Naso A, Carraro G, Wratten ML, Pagnin E, Bertipaglia L, et al. Effect of haemodiafiltration with online regeneration of ultrafiltrate on oxidative stress in dialysis patients. Nephrology Dialysis Transplantation. 2007; 22: 1413-1419. https://dx.doi.org/10.1093/ndt/gfl783. PMID: 17237480.

34. Calzavara P, Malagoli A, Zagatti R. [Comparative clinical study between standard hemodialysis, PHF (HDF on-line) and PHF AF (HDF on-line acetate free)]. Giornale Italiano di Nefrologia. 2004; 21 Suppl 30: S102-105. PMID: 15747290.

35. Canaud B. The Early Years of On-Line HDF: How Did It All Start? How Did We Get Here? In: Krick G, Ronco C, editors. On-Line Hemodiafiltration: The Journey and the Vision. 175. 2011. pp. 93-109.

36. Canaud B. High volume online hemodiafiltration: Technological aspects and clinical results. Artificial Organs. 2016; 40 (8): A2. PMID: 611868498.

37. Canaud B, Barbieri C, Marcelli D, Bellocchio F, Bowry S, Mari F, et al. Determination of optimal convective volume for improved patient outcome in a large incident dialysis cohort treated with online hemodiafiltration. Nephrology Dialysis Transplantation. 2014; 3): iii517. http://dx.doi.org/10.1093/ndt/gfu178. PMID: 71492958.

38. Canaud B, Barbieri C, Marcelli D, Bellocchio F, Bowry S, Mari F, et al. Optimal convection volume for improving patient outcomes in an international incident dialysis cohort treated with online hemodiafiltration. Kidney International. 2015; 88: 1108-1116. https://dx.doi.org/10.1038/ki.2015.139. PMID: 25945407.

39. Canaud B, Bayh I, Marcelli D, Ponce P, Merello JI, Gurevich K, et al. Superior survival of incident patients on high-volume online hemodiafiltration compared to high-flux hemodialysis. Nephrology Dialysis Transplantation. 2014; 29: iii32-33. PMID: CN-01010202 UPDATE.

40. Canaud B, Bosc JY, Cabrol L, Leray-Moragues H, Navino C, Verzetti G, et al. Urea as a marker of adequacy in hemodialysis: lesson from in vivo urea dynamics monitoring. Kidney International - Supplement. 2000; 76: S28-40. PMID: 10936797.

41. Canaud B, Bowry SK. Emerging clinical evidence on online hemodiafiltration: does volume of ultrafiltration matter? Blood Purification. 2013; 35: 55-62. https://dx.doi.org/10.1159/000345175. PMID: 23343547.

42. Canaud B, Chenine L, Henriet D, Leray H. Cross-membrane flux is a major factor influencing dialysis patient outcomes. Hemodialysis: From Basic Research to Clinical Trials. 2008; 161: 178-184. PMID: WOS:000257307700025.

43. Canaud B, Chenine L, Henriet D, Leray HM. Online hemodiafiltration: A multipurpose therapy for improving quality of renal replacement therapy. In: Ronco C, Cruz DN, editors. Hemodialysis: From Basic Research to Clinical Trials. 161. 2008. pp. 191-198.

44. Canaud B, Flavier JL, Argiles A, Stec F, QV NG, Bouloux C, et al. Hemodiafiltration with on-line production of substitution fluid: long-term safety and quantitative assessment of efficacy. Contributions to Nephrology. 1994; 108: 12-22. PMID: 8039393.

45. Canaud B, Levesque R, Krieter D, Desmeules S, Chalabi L, Moragues H, et al. On-line hemodiafiltration as routine treatment of end-stage renal failure: why pre- or mixed dilution mode is necessary in on-line hemodiafiltration today? Blood Purification. 2004; 22 Suppl 2: 40-48. https://dx.doi.org/10.1159/000081874. PMID: 15655323.

46. Canaud B, Morena M, Leray-Moragues H, Chalabi L, Cristol JP. Overview of clinical studies in hemodiafiltration: what do we need now ? Hemodialysis International. 2006; 10 Suppl 1: S5-S12. https://dx.doi.org/10.1111/j.1542-4758.2006.01183.x. PMID: 16441870.

47. Cantaluppi V, Cavallari C, Fonsato V, Dellepiane S, Marengo M, Migliori M, et al. Mixed on line hemodiafiltration modulates microRNA223 expression in circulating plasma extracellular vesicles: Protective role on endothelial dysfunction and vascular calcification in hemodialysis patients. Nephrology Dialysis Transplantation. 2016; 1): i32. http://dx.doi.org/10.1093/ndt/gfw131.2. PMID: 72325994.

48. Cerulli N, La Greca G, Ramello A, Schena F, Tetta C, Buades J, et al. The effect of hemodiafiltration with on-line endogenous reinfusion (on-line HFR) on anemia: Design of a European, open, randomised, multicentre trial. Journal of Nephrology. 2000; 13: 34-42.

49. Chancharoenthana W, Tiranathanagul K, Srisawat N, Susantitaphong P, Leelahavanichkul A, Praditpornsilpa K, et al. Enhanced vascular endothelial growth factor and inflammatory cytokine removal with online hemodiafiltration over high-flux hemodialysis in sepsis-related acute kidney injury patients. Therapeutic Apheresis & Dialysis: Official Peer-Reviewed Journal of the International Society for Apheresis, the Japanese Society for Apheresis, the Japanese Society for Dialysis Therapy. 2013; 17: 557-563. https://dx.doi.org/10.1111/1744-9987.12016. PMID: 24107284.

50. Chand DH. Atrial to compare hemodiafiltration v. conventional hemodialysis in pediatric patients with end-stage renal disease. Pediatric Nephrology. 2013; 28 (8): 1677. http://dx.doi.org/10.1007/s00467-013-2523-7. PMID: 71127907.

51. Chapdelaine I, Mostovaya IM, Blankestijn PJ, Bots ML, van den Dorpel MA, Levesque R, et al. Treatment policy rather than patient characteristics determines convection volume in online post-dilution hemodiafiltration. Blood Purification. 2014; 37: 229-237. https://dx.doi.org/10.1159/000362108. PMID: 24943743.

52. Chapdelaine I, Mostovaya IM, Blankestijn PJ, Bots ML, Van Den Dorpel MA, Nube MJ, et al. The type of vascular access is an independent determinant of the convective volume in post-dilution hemodiafiltration: Results from the convective transport study (CONTRAST). Nephrology Dialysis Transplantation. 2013; 1): i30. http://dx.doi.org/10.1093/ndt/gft159. PMID: 71075096.

53. Chazot C, Kirchgessner J, Pham J, Vo-Van C, Lorriaux C, Hurot JM, et al. Effect of Membrane Permeability on Cardiovascular Risk Factors and beta 2m Plasma Levels in Patients on Long-Term Haemodialysis: A Randomised Crossover Trial. Nephron. 2015; 129: 269-275. 10.1159/000380767. PMID: WOS:000353721100006.

54. Chevalier L, Tielemans C, Debelle F, Vandervelde D, Fumeron C, Mandart L, et al. Isonatric Dialysis Biofeedback in Hemodiafiltration with Online Regeneration of Ultrafiltrate in Hypertensive Hemodialysis Patients: A Randomized Controlled Study. Blood Purification. 2016; 41: 87-93. https://dx.doi.org/10.1159/000441967. PMID: 26580275.

55. Cogliati P. [Thermal sensor and on-line hemodiafiltration]. Giornale Italiano di Nefrologia. 2005; 22 Suppl 31: S111-116. PMID: 15786382.

56. Coll E, Perez-Garcia R, Martin de Francisco AL, Galceran J, Garcia-Osuna R, Martin-Malo A, et al. [Acetate-free on-line PHF: how to improve hyperacetatemia and haemodynamic tolerance]. Nefrologia. 2009; 29: 156-162. https://dx.doi.org/10.3265/Nefrologia.2009.29.2.4969.en.full. PMID: 19396322.

57. Combarnous F, Tetta C, Chapuis Cellier C, Wratten ML, Custaud MA, De Catheu T, et al. Albumin loss in on-line hemodiafiltration. International Journal of Artificial Organs. 2002; 25: 203-209. PMID: 34456096.

58. Cornelis T, van der Sande FM, Eloot S, Cardinaels E, Bekers O, Damoiseaux J, et al. Acute hemodynamic response and uremic toxin removal in conventional and extended hemodialysis and hemodiafiltration: a randomized crossover study. American journal of kidney diseases : the official journal of the National Kidney Foundation. 2014; 64: 247-256. PMID: CN-00998735 UPDATE.

59. Cross J, Davenport A. Does online hemodiafiltration lead to reduction in trace elements and vitamins? Hemodialysis International. 2011; 15: 509-514. https://dx.doi.org/10.1111/j.1542-4758.2011.00580.x. PMID: 22111819.

60. Davenport A. New Dialysis Technology and Biocompatible Materials. Contributions to Nephrology. 2017; 189: 130-136. https://dx.doi.org/10.1159/000450739. PMID: 27951560.

61. Davenport A, Peters SA, Bots ML, Canaud B, Grooteman MP, Asci G, et al. Higher convection volume exchange with online hemodiafiltration is associated with survival advantage for dialysis patients: the effect of adjustment for body size. Kidney International. 2016; 89: 193-199. https://dx.doi.org/10.1038/ki.2015.264. PMID: 26352299.

62. de Francisco ALM, Pinera C. Challenges and future of renal replacement therapy. Hemodialysis International. 2006; 10: S19-S23. http://dx.doi.org/10.1111/j.1542-4758.2006.01185.x. PMID: 43263303.

63. de Sequera P, Albalate M, Perez-Garcia R, Corchete E, Puerta M, Ortega M, et al. A comparison of the effectiveness of two online haemodiafiltration modalities: mixed versus post-dilution. Nefrologia. 2013; 33: 779-787. https://dx.doi.org/10.3265/Nefrologia.pre2013.Sep.12223. PMID: 24241365.

64. den Hoedt CH, Bots ML, Grooteman MP, Mazairac AH, Penne EL, van der Weerd NC, et al. Should we still focus that much on cardiovascular mortality in end stage renal disease patients? The CONvective TRAnsport STudy. PLoS ONE [Electronic Resource]. 2013; 8: e61155. https://dx.doi.org/10.1371/journal.pone.0061155. PMID: 23620729.

65. den Hoedt CH, Bots ML, Grooteman MP, van der Weerd NC, Mazairac AH, Penne EL, et al. Online hemodiafiltration reduces systemic inflammation compared to low-flux hemodialysis. Kidney International. 2014; 86: 423-432. https://dx.doi.org/10.1038/ki.2014.9. PMID: 24552852.

66. den Hoedt CH, Bots ML, Grooteman MP, van der Weerd NC, Penne EL, Mazairac AH, et al. Clinical predictors of decline in nutritional parameters over time in ESRD. Clinical Journal of The American Society of Nephrology: CJASN. 2014; 9: 318-325. https://dx.doi.org/10.2215/CJN.04470413. PMID: 24458074.

67. den Hoedt CH, Grooteman MP, Bots ML, Blankestijn PJ, van der Tweel I, van der Weerd NC, et al. The Effect of Online Hemodiafiltration on Infections: Results from the CONvective TRAnsport STudy. PLoS ONE [Electronic Resource]. 2015; 10: e0135908. https://dx.doi.org/10.1371/journal.pone.0135908. PMID: 26288091.

68. Dhondt A, Pauwels R, Devreese K, Eloot S, Glorieux G, Vanholder R. Where and When To Inject Low Molecular Weight Heparin in Hemodiafiltration? A Cross Over Randomised Trial. Plos one. 2015; 10: e0128634. PMID: CN-01257540.

69. Ding F, Ahrenholz P, Winkler RE, Ramlow W, Tiess M, Michelsen A, et al. Online hemodiafiltration versus acetate-free biofiltration: a prospective crossover study. Artificial Organs. 2002; 26: 169-180. PMID: 11879247.

70. Dolley-Hitze T, Dubois N, Stanescu C, Bahon-Riedinger I, Hamel D, Lombart ML, et al. A new asymetric cellulose triacetate membrane is effecient and safe in online post-dilution hemodiafiltration. Nephrology Dialysis Transplantation. 2017; 32 (Supplement 3): iii615. http://dx.doi.org/10.1093/ndt/gfx175. PMID: 617302519.

71. Dolley-Hitze T, Hamel D, Lombart ML. Citric acid and acetate free based dialysate in online hemodiafiltration post-dilution allows heparin free sessions. Nephrology Dialysis Transplantation. 2014; 29: iii220. PMID: CN-01060950 NEW.

72. Donadio C, Tognotti D. High-flux dialysis: Clinical, biochemical, and proteomic comparison with on-line haemodiafiltration. Nephrology. 2016; 21 (Supplement 2): 181-182. http://dx.doi.org/10.1111/nep.12888. PMID: 612312738.

73. Dusilova Sulkova S, Lopot F. Effectiveness and safety: Document of European initiative on-line haemodiafiltration - Definiton, eudial and comments on results of prospective controlled clinical trials. [Czech]. Aktuality v Nefrologii. 2014; 20: 43-49. PMID: 372833599.

74. Ethier I, Auger D, Beaulieu M, Cardinal H, Levesque R. Evolution of high-sensitivity troponin-T in patients undergoing high efficiency on-line hemodiafiltration versus conventional low-flux hemodialysis. Nephrology Dialysis Transplantation Conference: 52nd ERA EDTA Congress London United Kingdom Conference Start. 2015; 30: iii282. PMID: CN-01173025 NEW.

75. Ethier I, Cardinal H, Beaulieu M, Grooteman MPC, Nube MJ, Levesque R, et al. Evolution of high sensitivity troponin-T in patients undergoing high efficiency on-line hemodiafiltration versus conventional low-flux hemodialysis. European Heart Journal. 2015; 1): 924-925. http://dx.doi.org/10.1093/eurheartj/ehv401. PMID: 72022203.

76. Fan QC, Yan YC, Gu LY, He LQ, Chen N, Jiang GR, et al. Prognostic Value of the Delivery Dialysis Dose on Twice-Weekly Hemodialysis Patients. American Journal of Nephrology. 2017; 45: 273-282. 10.1159/000453043. PMID: WOS:000395936300010.

77. Ferreira A, Pinto B, Navarro D, Aniceto J, Neves P, Ponce P. Iron-based phosphate binders in on-line hemodiafiltration patients-a portuguese experience. Nephrology Dialysis Transplantation. 2017; 32 (Supplement 3): iii684. http://dx.doi.org/10.1093/ndt/gfx179. PMID: 617290488.

78. Ficheux A, Gayrard N, Duranton F, Guzman C, Szwarc I, Vetromile F, et al. A reliable method to assess the water permeability of a dialysis system: the global ultrafiltration coefficient. Nephrology Dialysis Transplantation. 2017; 32: 364-370. 10.1093/ndt/gfw370. PMID: WOS:000397027900022.

79. Fischbach M, Terzic J, Laugel V, Dheu C, Menouer S, Helms P, et al. Daily on-line haemodiafiltration: a pilot trial in children. Nephrology Dialysis Transplantation. 2004; 19: 2360-2367. https://dx.doi.org/10.1093/ndt/gfh403. PMID: 15266034.

80. Francisco RC, Aloha M, Ramon PS. Effects of high-efficiency postdilution online hemodiafiltration and high-flux hemodialysis on serum phosphorus and cardiac structure and function in patients with end-stage renal disease. International Urology & Nephrology. 2013; 45: 1373-1378. https://dx.doi.org/10.1007/s11255-012-0324-8. PMID: 23143753.

81. Garcia-Fernandez N, Lavilla FJ, Rocha E, Purroy A. Haemostatic changes in systemic inflammatory response syndrome during continuous renal replacement therapy. Journal of nephrology. 2000; 13: 282-289. PMID: CN-00329879 UPDATE.

82. Gayrard N, Ficheux A, Duranton F, Guzman C, Szwarc I, Vetromile F, et al. Consequences of increasing convection onto patient care and protein removal in hemodialysis. PLoS ONE [Electronic Resource]. 2017; 12: e0171179. https://dx.doi.org/10.1371/journal.pone.0171179. PMID: 28166268.

83. Ghigolea AB, Gherman-Caprioara M, Moldovan AR. Arterial stiffness: hemodialysis versus hemodiafiltration. Clujul Medical. 2017; 90: 166-170. https://dx.doi.org/10.15386/cjmed-699. PMID: 28559700.

84. Gonzalez-Diez B, Cavia M, Torres G, Abaigar P, Camarero V, Muniz P. The effects of 1-year treatment with a haemodiafiltration with on-line regeneration of ultrafiltrate (HFR) dialysis on biomarkers of oxidative stress in patients with chronic renal failure. Molecular Biology Reports. 2012; 39: 629-634. https://dx.doi.org/10.1007/s11033-011-0780-8. PMID: 21603859.

85. Grooteman MP, van den Dorpel MA, Bots ML, Penne EL, van der Weerd NC, Mazairac AH, et al. Effect of online hemodiafiltration on all-cause mortality and cardiovascular outcomes. Journal of the American Society of Nephrology. 2012; 23: 1087-1096. https://dx.doi.org/10.1681/ASN.2011121140. PMID: 22539829.

86. Gross M, Maierhofer A, Tetta C, Senecal L, Canaud B. Online clearance measurement in high-efficiency hemodiafiltration. Kidney International. 2007; 72: 1550-1553. https://dx.doi.org/10.1038/sj.ki.5002551. PMID: 17882147.

87. Grundstrom G, Christensson A, Alquist M, Nilsson LG, Segelmark M. Replacement of acetate with citrate in dialysis fluid: a randomized clinical trial of short term safety and fluid biocompatibility. BMC Nephrology. 2013; 14: 216. https://dx.doi.org/10.1186/1471-2369-14-216. PMID: 24103587.

88. Grzegorzewska AE, Cieszynski K, Kaczmarek A, Wiesiolowska A. High sensitivity cardiac troponin T (HS-CTNT) in continuous ambulatory peritoneal dialysis patients (CAPD) and extracorporeal dialysis patients. Peritoneal Dialysis International. 2010; 2): S129. PMID: 71928177.

89. Guery B, Alberti C, Servais A, Harrami E, Bererhi L, Zins B, et al. Hemodialysis without systemic anticoagulation: a prospective randomized trial to evaluate 3 strategies in patients at risk of bleeding. PLoS ONE [Electronic Resource]. 2014; 9: e97187. https://dx.doi.org/10.1371/journal.pone.0097187. PMID: 24825343.

90. Hazelbag CM, Peters SAE, Blankestijn PJ, Bots ML, Canaud B, Davenport A, et al. The importance of considering competing treatment affecting prognosis in the evaluation of therapy in trials: the example of renal transplantation in hemodialysis trials. Nephrology Dialysis Transplantation. 2017; 32: ii31-ii39. https://dx.doi.org/10.1093/ndt/gfw458. PMID: 28339826.

91. Herrington W, Haynes R, Staplin N, Emberson J, Baigent C, Landray M. Evidence for the prevention and treatment of stroke in dialysis patients. Seminars in Dialysis. 2015; 28: 35-47. https://dx.doi.org/10.1111/sdi.12281. PMID: 25040468.

92. Hudier L, Decaux O, Haddj-Elmrabet A, Mandart L, Lino-Daniel M, Bridoux F, et al. Impact of intensive daily haeodialysis on renal recovery of cast nephropathies: A retrospective study. Nephrology Dialysis Transplantation. 2012; 2): ii361. http://dx.doi.org/10.1093/ndt/gfs235. PMID: 70766369.

93. Jardine M, Krishnan A, Gallagher M, Snelling P, Hawley C, Pussell B, et al. Uraemic neuropathy remains ubiquitious: Rationale and feasibility of the finesse (filtration in the neuropathy of end stage kidney disease symptom evolution) trial. Nephrology. 2010; 4): 88. http://dx.doi.org/10.1111/j.1440-1797.2010.01377.x. PMID: 70467239.

94. Jeloka TK. Online hemodiafiltration - A systematic review. Clinical Queries: Nephrology. 2013; 2: 145-147. http://dx.doi.org/10.1016/j.cqn.2013.11.004. PMID: 370576193.

95. Jia P, Jin W, Teng J, Zhang H, Zou J, Liu Z, et al. Acute Effects of Hemodiafiltration Versus Conventional Hemodialysis on Endothelial Function and Inflammation: A Randomized Crossover Study. Medicine. 2016; 95: e3440. https://dx.doi.org/10.1097/MD.0000000000003440. PMID: 27100440.

96. Joyeux V, Sijpkens Y, Haddj-Elmrabet A, Bijvoet AJ, Nilsson LG. Optimized convective transport with automated pressure control in on-line postdilution hemodiafiltration. International Journal of Artificial Organs. 2008; 31: 928-936. PMID: 19089794.

97. Kalousova M, Kielstein JT, Hodkova M, Zima T, Dusilova-Sulkova S, Martens-Lobenhoffer J, et al. No benefit of hemodiafiltration over hemodialysis in lowering elevated levels of asymmetric dimethylarginine in ESRD patients. Blood Purification. 2006; 24: 439-444. https://dx.doi.org/10.1159/000095360. PMID: 16940714.

98. Kang SW, Kim HJ, Park SJ, Kim TH, Kim YH. The effect of on-line hemodiafiltration on dry weight adjustment with bio-impedance: Comparative study between conventional hemodialysis and on-line hemodiafiltration. Peritoneal Dialysis International. 2010; 2): S136. PMID: 71928206.

99. Kanter J, Puerta MC, Garcia RP, Gomez JM, Jofre R, Rodriguez PB. [On-line sequential hemodiafiltration (HDF-OL-S): a new therapeutic option]. Nefrologia. 2008; 28: 433-438. PMID: 18662152.

100. Kapun S. Does online hemodiafiltration (oHDF) improve the outcome of chronic dialysis patients in comparison with high-flux hemodialysis (HD). Acta Medica Croatica. 2014; 2): 254.

101. Kapun S. Does online hemodiafiltration (oHDF) improve the outcome of chronic dialysis patients in comparison with high-flux hemodialysis (HD). [Croatian]. Acta Medica Croatica. 2014; 68: 55-57. PMID: 606160793.

102. Kawanishi H. What can we expect from on-line hemodiafiltration? Blood Purification. 2013; 35 Suppl 1: 1-5. https://dx.doi.org/10.1159/000346217. PMID: 23466370.

103. Kawanishi H. [On-line hemodiafiltration (HDF)]. Nippon Jinzo Gakkai Shi Japanese Journal of Nephrology. 2013; 55: 523-528. PMID: 23819378.

104. Kim S, Oh KH, Chin HJ, Na KY, Kim YS, Chae DW, et al. Effective removal of leptin via hemodiafiltration with on-line endogenous reinfusion therapy. Clinical Nephrology. 2009; 72: 442-448. PMID: 19954721.

105. Knehtl M, Jakopin E, Bevc S, Ekart R, Hojs R. The effect of haemodialysis and post-dilution haemodiafiltration on platelet closure time in patients with end-stage renal disease. Nephrology Dialysis Transplantation. 2017; 32 (Supplement 3): iii621-iii622. http://dx.doi.org/10.1093/ndt/gfx175. PMID: 617302555.

106. Koda Y, Aoike I, Hasegawa S, Osawa Y, Nakagawa Y, Iwabuchi F, et al. Feasibility of intermittent back-filtrate infusion hemodiafiltration to reduce intradialytic hypotension in patients with cardiovascular instability: a pilot study. Clinical & Experimental Nephrology. 2017; 21: 324-332. https://dx.doi.org/10.1007/s10157-016-1270-z. PMID: 27125432.

107. Kosmadakis G, Correia EDC, Albaret J, Somda F, Aguilera D. Comparison of the hemodynamic tolerance and the biological parameters of four acetate-free hemodialysis methods. Nephrologie et Therapeutique. 2017; 13: 532-536. https://dx.doi.org/10.1016/j.nephro.2017.03.002. PMID: 29133076.

108. Kosmadakis G, Da Costa Correia E, Somda F, Aubailly L, Piffaut MC, Aguilera D. Comparison of the hemodynamic tolerance and the biological parameters of four acetate-free dialysis methods in a group of thrice weekly hemodialysis patients. Nephrology Dialysis Transplantation Conference: 52nd ERA EDTA Congress London United Kingdom Conference Start. 2015; 30: iii564. PMID: CN-01172772 NEW.

109. Krieter DH, Collins G, Summerton J, Spence E, Moragues HL, Canaud B. Mid-dilution on-line haemodiafiltration in a standard dialyser configuration. Nephrology Dialysis Transplantation. 2005; 20: 155-160. https://dx.doi.org/10.1093/ndt/gfh520. PMID: 15522903.

110. Krieter DH, Falkenhain S, Chalabi L, Collins G, Lemke HD, Canaud B. Clinical cross-over comparison of mid-dilution hemodiafiltration using a novel dialyzer concept and post-dilution hemodiafiltration. Kidney International. 2005; 67: 349-356. https://dx.doi.org/10.1111/j.1523-1755.2005.00088.x. PMID: 15610261.

111. Krieter DH, Hackl A, Rodriguez A, Chenine L, Moragues HL, Lemke HD, et al. Protein-bound uraemic toxin removal in haemodialysis and post-dilution haemodiafiltration. Nephrology Dialysis Transplantation. 2010; 25: 212-218. https://dx.doi.org/10.1093/ndt/gfp437. PMID: 19755476.

112. Krieter DH, Hunn E, Morgenroth A, Lemke HD, Wanner C. Matching efficacy of online hemodiafiltration in simple hemodialysis mode. Artificial Organs. 2008; 32: 903-909. https://dx.doi.org/10.1111/j.1525-1594.2008.00652.x. PMID: 19133017.

113. Krieter DH, Korner T, Devine E, Ruth M, Jankowski J, Wanner C, et al. Pilot trialon ionic strength hemodiafiltration, a novel dialysis technique for increased protein bound toxin removal. Nephrology Dialysis Transplantation. 2014; 29: iii211-212. PMID: CN-01010194 UPDATE.

114. Kron J, Schneditz D, Leimbach T, Aign S, Kron S. A simple and feasible method to determine absolute blood volume in hemodialysis patients in clinical practice. Blood Purification. 2014; 38: 180-187. https://dx.doi.org/10.1159/000368157. PMID: 25531533.

115. Kurtkoti J, Bose B, Hiremagalur B, Sun J, Cochrane T. Arterial line versus venous line administration of lowmolecular weight heparin, enoxaparin for prevention of thrombosis in the extracorporeal blood circuit of patients on haemodialysis or haemodiafiltration-a randomized cross-over trial. Nephrology Dialysis Transplantation Conference: 52nd ERA EDTA Congress London United Kingdom Conference Start. 2015; 30: iii251. PMID: CN-01173056 NEW.

116. Kurtkoti J, Bose B, Hiremagalur B, Sun J, Cochrane T. Arterial line versus venous line administration of low molecular weight heparin, enoxaparin for prevention of thrombosis in the extracorporeal blood circuit of patients on haemodialysis or haemodiafiltration: a randomized cross-over trial. Nephrology (carlton, vic). 2016; 21: 663-668. PMID: CN-01177295.

117. Lameire N, Van Biesen W, Vanholder R. Did 20 years of technological innovations in hemodialysis contribute to better patient outcomes? Clinical Journal of the American Society of Nephrology. 2009; 4: S30-S40. http://dx.doi.org/10.2215/CJN.04000609. PMID: 358082037.

118. Laskin BL, Huang GX, King E, Geary DF, Licht C, Metlay JP, et al. Short, frequent, 5-days-per-week, in-center hemodialysis versus 3-days-per week treatment: a randomized crossover pilot trial through the Midwest Pediatric Nephrology Consortium. Pediatric Nephrology. 2017; 32: 1423-1432. 10.1007/s00467-017-3656-x. PMID: WOS:000404238200019.

119. Lee K, Mun CH, Min BG, Won YS. A Dual-Chambered Hemodialyzer for Convection-Enhanced Hemodialysis. Artificial Organs. 2012; 36: E78-E82. 10.1111/j.1525-1594.2011.01402.x. PMID: WOS:000301112600008.

120. Lee K, Pino CJ, Humes HD. Substitution-free hemodiafiltration. ASAIO Journal. 2012; 58: 514-521. https://dx.doi.org/10.1097/MAT.0b013e318260c5bb. PMID: 22951895.

121. Lema LV, Ravina AR. Effectiveness and safety of different hemodialysis modalities: a review. Journal of Nephrology. 2007; 20: 525-542. PMID: WOS:000254723800006.

122. Levesque R, Marcelli D, Cardinal H, Caron ML, Grooteman M, Bots M, et al. Cost-effectiveness analysis of high-efficiency hemodiafiltration vs. Low-flux hemodialysis based on the canadian arm of the contrast study. Nephrology Dialysis Transplantation. 2015; 30: iii331. PMID: CN-01139468 NEW.

123. Levesque R, Marcelli D, Cardinal H, Caron ML, Grooteman MP, Bots ML, et al. Cost-Effectiveness Analysis of High-Efficiency Hemodiafiltration Versus Low-Flux Hemodialysis Based on the Canadian Arm of the CONTRAST Study. Applied Health Economics & Health Policy. 2015; 13: 647-659. https://dx.doi.org/10.1007/s40258-015-0179-0. PMID: 26071951.

124. Li X, Li M, Liu T, Li L, Duan L, Li Y, et al. Hemofiltration or hemodiafiltration with on-line production of substitution fluid: clinical observation of safety and effectiveness. Chinese Medical Journal. 1997; 110: 520-525. PMID: 9594209.

125. Lin CL, Huang CC, Chang CT, Wu MS, Hung CC, Chien CC, et al. Clinical improvement by increased frequency of on-line hemodialfiltration. Renal Failure. 2001; 23: 193-206. PMID: 11417951.

126. Lin CL, Huang CC, Yu CC, Yang HY, Chuang FR, Yang CW. Reduction of advanced glycation end product levels by on-line hemodiafiltration in long-term hemodialysis patients. American Journal of Kidney Diseases. 2003; 42: 524-531. PMID: 12955680.

127. Lin CL, Yang CW, Chiang CC, Chang CT, Huang CC. Long-term on-line hemodiafiltration reduces predialysis beta-2-microglobulin levels in chronic hemodialysis patients. Blood Purification. 2001; 19: 301-307. https://dx.doi.org/46958. PMID: 11244190.

128. Lips L, van Zuijdewijn C, ter Wee PM, Bots ML, Blankestijn PJ, van den Dorpel MA, et al. Serum sclerostin: relation with mortality and impact of hemodiafiltration. Nephrology Dialysis Transplantation. 2017; 32: 1217-1223. 10.1093/ndt/gfw246. PMID: WOS:000405421100017.

129. Liu S, Zhang DL, Guo W, Cui WY, Liu WH. Left ventricular mass index and aortic arch calcification score are independent mortality predictors of maintenance hemodialysis patients. Hemodialysis International. 2012; 16: 504-511. https://dx.doi.org/10.1111/j.1542-4758.2012.00700.x. PMID: 22520823.

130. Locatelli F, Altieri P, Andrulli S, Bolasco P, Sau G, Pedrini LA, et al. Hemofiltration and hemodiafiltration reduce intradialytic hypotension in ESRD. Journal of the American Society of Nephrology. 2010; 21: 1798-1807. https://dx.doi.org/10.1681/ASN.2010030280. PMID: 20813866.

131. Locatelli F, Altieri P, Andrulli S, Sau G, Bolasco P, Pedrini LA, et al. Predictors of haemoglobin levels and resistance to erythropoiesis-stimulating agents in patients treated with low-flux haemodialysis, haemofiltration and haemodiafiltration: results of a multicentre randomized and controlled trial. Nephrology Dialysis Transplantation. 2012; 27: 3594-3600. https://dx.doi.org/10.1093/ndt/gfs117. PMID: 22622452.

132. Locatelli F, Altieri P, Andrulli S, Sau G, Bolasco P, Pedrini LA, et al. Phosphate levels in patients treated with low-flux haemodialysis, pre-dilution haemofiltration and haemodiafiltration: post hoc analysis of a multicentre, randomized and controlled trial. Nephrology Dialysis Transplantation. 2014; 29: 1239-1246. https://dx.doi.org/10.1093/ndt/gfu031. PMID: 24557989.

133. Locatelli F, Andrulli S, Di Filippo S, Redaelli B, Mangano S, Navino C, et al. Effect of on-line conductivity plasma ultrafiltrate kinetic modeling on cardiovascular stability of hemodialysis patients. Kidney International. 1998; 53: 1052-1060. https://dx.doi.org/10.1111/j.1523-1755.1998.00844.x. PMID: 9551417.

134. Locatelli F, Canaud B. Dialysis adequacy today: a European perspective. Nephrology Dialysis Transplantation. 2012; 27: 3043-3048. https://dx.doi.org/10.1093/ndt/gfs184. PMID: 22743335.

135. Locatelli F, Del Vecchio L, Andrulli S. Dialysis: Its role in optimizing recombinant erythropoietin treatment. Nephrology Dialysis Transplantation. 2001; 16: 29-35. PMID: 33062120.

136. Locatelli F, Del Vecchio L, La Milia V. Haemodiafiltration at Higher Volumes and Patient Survival. Contributions to Nephrology. 2017; 189: 1-8. https://dx.doi.org/10.1159/000450632. PMID: 27951544.

137. Locatelli F, Di Filippo S, Pozzoni P. A critical assessment of uremia research. Blood Purification. 2006; 24: 71-76. https://dx.doi.org/10.1159/000089441. PMID: 16361845.

138. Locatelli F, Manzoni C, Del Vecchio L, Cavalli A, Pontoriero G. Recent trials on hemodiafiltration. Contributions to Nephrology. 2011; 171: 92-100. https://dx.doi.org/10.1159/000327202. PMID: 21625096.

139. Locatelli F, Manzoni C, Di Filippo S. The importance of convective transport. Kidney International. 2002; 61: S115-S120. 10.1046/j.1523-1755.61.s80.21.x. PMID: WOS:000175117600023.

140. Locatelli F, Manzoni C, Pozzoni P, Pontoriero G, Di Filippo L. [Clinical dialysis: new problems and new prospects]. Giornale Italiano di Nefrologia. 2004; 21: 156-164. PMID: 15351950.

141. Locatelli F, Manzoni C, Vigano S, Cavalli A, Di Filippo S. Hemodiafiltration - state of the art. Contributions to Nephrology. 2011; 168: 5-18. https://dx.doi.org/10.1159/000321740. PMID: 20938121.

142. Locatelli F, Stefoni S, Petitclerc T, Coli L, Di Filippo S, Andrulli S, et al. Effect of a plasma sodium biofeedback system applied to HFR on the intradialytic cardiovascular stability. Results from a randomized controlled study. Nephrology Dialysis Transplantation. 2012; 27: 3935-3942. http://dx.doi.org/10.1093/ndt/gfs091. PMID: 365995368.

143. Locatelli F, Violo L, Longhi S, Del Vecchio L. Current Evidence in Haemodiafiltration. Blood Purification. 2015; 40 Suppl 1: 24-29. https://dx.doi.org/10.1159/000437410. PMID: 26344510.

144. Lornoy W, Becaus I, Billiouw JM, Sierens L, van Malderen P. Remarkable removal of beta-2-microglobulin by on-line hemodiafiltration.[Erratum appears in Am J Nephrol 2000 Mar-Apr;20(2):168]. American Journal of Nephrology. 1998; 18: 105-108. https://dx.doi.org/13317. PMID: 9569951.

145. Lornoy W, Becaus I, Billiouw JM, Sierens L, Van Malderen P, D'Haenens P. On-line haemodiafiltration. Remarkable removal of beta2-microglobulin. Long-term clinical observations. Nephrology Dialysis Transplantation. 2000; 15 Suppl 1: 49-54. PMID: 10737167.

146. Lornoy W, De Meester J, Becaus I, Billiouw JM, Van Malderen PA, Van Pottelberge M. Impact of convective flow on phosphorus removal in maintenance hemodialysis patients. Journal of Renal Nutrition. 2006; 16: 47-53. https://dx.doi.org/10.1053/j.jrn.2005.10.008. PMID: 16414441.

147. Macias N, Santos A, Vega A, Abad S, Cedeno SA, Lopez Gomez JM. The effect of body composition in the efficacy of convective transport in on-line hemodiafiltration. Nephrology Dialysis Transplantation. 2016; 1): i66. http://dx.doi.org/10.1093/ndt/gfw145.2. PMID: 72326065.

148. Macias N, Santos Garcia A, Vega Martinez A, Abad Estebanez S, Goicoechea Diezhandino M, Lopez Gomez JM. Importance of Body Water in the Efficacy of Convective Solute Transport in Online Hemodiafiltration. Therapeutic Apheresis & Dialysis: Official Peer-Reviewed Journal of the International Society for Apheresis, the Japanese Society for Apheresis, the Japanese Society for Dialysis Therapy. 2017; 21: 88-95. https://dx.doi.org/10.1111/1744-9987.12486. PMID: 28093894.

149. Maduell F. Is There an 'Optimal Dose' of Hemodiafiltration? Blood Purification. 2015; 40 Suppl 1: 17-23. https://dx.doi.org/10.1159/000437409. PMID: 26344509.

150. Maduell F, Arias M, Duran CE, Vera M, Fontsere N, Azqueta M, et al. Nocturnal, every-other-day, online haemodiafiltration: an effective therapeutic alternative. Nephrology Dialysis Transplantation. 2012; 27: 1619-1631. https://dx.doi.org/10.1093/ndt/gfr491. PMID: 21931125.

151. Maduell F, Arias-Guillen M, Fontsere N, Ojeda R, Rico N, Vera M, et al. Elimination of large uremic toxins by a dialyzer specifically designed for high-volume convective therapies. Blood Purification. 2014; 37: 125-130. https://dx.doi.org/10.1159/000358214. PMID: 24662288.

152. Maduell F, Garcia H, Hdez-Jaras I, Calvo C, Navarro V. Effect of blood flow (Q8) and infusion flow rate (Qi) on efficacy of on- line hemodiafiltration. [Spanish]. Nefrologia. 1999; 19: 31-38. PMID: 29148594.

153. Maduell F, Moreso F, Mora-Macia J, Pons M, Ramos R, Carreras J, et al. ESHOL study reanalysis: All-cause mortality considered by competing risks and time-dependent covariates for renal transplantation. Nefrologia. 2016; 36: 156-163. https://dx.doi.org/10.1016/j.nefro.2015.10.007. PMID: 26672890.

154. Maduell F, Moreso F, Pons M, Ramos R, Mora-Macia J, Carreras J, et al. High-efficiency postdilution online hemodiafiltration reduces all-cause mortality in hemodialysis patients.[Erratum appears in J Am Soc Nephrol. 2014 May;25(5):1130]. Journal of the American Society of Nephrology. 2013; 24: 487-497. https://dx.doi.org/10.1681/ASN.2012080875. PMID: 23411788.

155. Maduell F, Moreso F, Pons M, Ramos R, Mora-Macia J, Foraster A, et al. Design and patient characteristics of ESHOL study, a Catalonian prospective randomized study. Journal of Nephrology. 2011; 24: 196-202. PMID: 20602331.

156. Maduell F, Navarro V, Rius A, Torregrosa E, Sanchez JJ, Saborit ML, et al. [Improvement of nutritional status in patients with short daily on-line hemodiafiltration]. Nefrologia. 2004; 24: 60-66. PMID: 15083959.

157. Maduell F, Ojeda R, Arias-Guillen M, Fontsere N, Vera M, Masso E, et al. Optimization of dialysate flow in on-line hemodiafiltration. Nefrologia. 2015; 35: 473-478. https://dx.doi.org/10.1016/j.nefro.2015.06.019. PMID: 26306957.

158. Maduell F, Ojeda R, Rodas L, Rico N, Fontsere N, Arias M, et al. On-line haemodiafiltration with auto-substitution: assessment of blood flow changes on convective volume and efficiency. Nefrologia. 2015; 35: 50-57. https://dx.doi.org/10.3265/Nefrologia.pre2014.Sep.12726. PMID: 25611833.

159. Maheshwari V, Samavedham L, Rangaiah GP, Loy Y, Ling LH, Sethi S, et al. Comparison of toxin removal outcomes in online hemodiafiltration and intra-dialytic exercise in high-flux hemodialysis: a prospective randomized open-label clinical study protocol. BMC Nephrology. 2012; 13: 156. https://dx.doi.org/10.1186/1471-2369-13-156. PMID: 23176731.

160. Mandolfo S, Borlandelli S, Imbasciati E, Badalamenti S, Graziani G, Sereni L, et al. Pilot study to assess increased dialysis efficiency in patients with limited blood flow rates due to vascular access problems. Hemodialysis International. 2008; 12: 55-61. https://dx.doi.org/10.1111/j.1542-4758.2008.00241.x. PMID: 18271842.

161. Marcelli D, Bayh I, Merello JI, Ponce P, Heaton A, Kircelli F, et al. Dynamics of the erythropoiesis stimulating agent resistance index in incident hemodiafiltration and high-flux hemodialysis patients. Kidney International. 2016; 90: 192-202. 10.1016/j.kint.2016.03.009. PMID: WOS:000377929400028.

162. Marcinkowski W, Drozdz M, Milkowski A, Rydzynska T, Prystacki T, August R, et al. The results of multicenter study comparing effects of different renal replacement therapy modalities (online hemodiafiltration vs. low-flux hemodialysis). Nephrology Dialysis Transplantation. 2012; 2): ii208. http://dx.doi.org/10.1093/ndt/gfs224. PMID: 70765907.

163. Marinez de Francisco AL, Ghezzi PM, Brendolan A, Fiorini F, La Greca G, Ronco C, et al. Hemodiafiltration with online regeneration of the ultrafiltrate. Kidney International - Supplement. 2000; 76: S66-71. PMID: 10936801.

164. Marshall MR, Ma T, Galler D, Rankin AP, Williams AB. Sustained low-efficiency daily diafiltration (SLEDD-f) for critically ill patients requiring renal replacement therapy: towards an adequate therapy. Nephrology Dialysis Transplantation. 2004; 19: 877-884. https://dx.doi.org/10.1093/ndt/gfg625. PMID: 15031344.

165. Masakane I. Choice of modality with the use of high-performance membrane and evaluation for clinical effects. Contributions to Nephrology. 2011; 173: 84-94. https://dx.doi.org/10.1159/000328959. PMID: 21865780.

166. Masakane I, Kikuchi K, Wada A, Hamano T, Tomo T. Pre-dilution on-line HDF has survival advantage on choronic dialysis patients: From the Japanese nationwide registry. Nephrology Dialysis Transplantation. 2016; 1): i66. http://dx.doi.org/10.1093/ndt/gfw145.1. PMID: 72326064.

167. Matos JF, Peralta P, Felix C, Carvalho H, Pinto B, Ponce P. Treatment related predictors and relation to substitution volume and dialysis dose on patients under online haemodifiltration. Nephrology Dialysis Transplantation. 2017; 32 (Supplement 3): iii79. http://dx.doi.org/10.1093/ndt/gfx127. PMID: 617291143.

168. Matsuyama K, Tomo T, Kadota J. Acetate-free blood purification can impact improved nutritional status in hemodialysis patients. Journal of Artificial Organs. 2011; 14: 112-119. https://dx.doi.org/10.1007/s10047-010-0551-7. PMID: 21336818.

169. Mazairac AH, Blankestijn PJ, Grooteman MP, Penne EL, van der Weerd NC, den Hoedt CH, et al. The cost-utility of haemodiafiltration versus haemodialysis in the Convective Transport Study. Nephrology Dialysis Transplantation. 2013; 28: 1865-1873. https://dx.doi.org/10.1093/ndt/gft045. PMID: 23766337.

170. Meert N, Eloot S, Schepers E, Lemke HD, Dhondt A, Glorieux G, et al. Comparison of removal capacity of two consecutive generations of high-flux dialysers during different treatment modalities. Nephrology Dialysis Transplantation. 2011; 26: 2624-2630. https://dx.doi.org/10.1093/ndt/gfq803. PMID: 21310741.

171. Meert N, Eloot S, Waterloos MA, Van Landschoot M, Dhondt A, Glorieux G, et al. Effective removal of protein-bound uraemic solutes by different convective strategies: a prospective trial. Nephrology Dialysis Transplantation. 2009; 24: 562-570. https://dx.doi.org/10.1093/ndt/gfn522. PMID: 18809977.

172. Melo NC, Moyses RM, Elias RM, Castro MC. Reprocessing high-flux polysulfone dialyzers does not negatively impact solute removal in short-daily online hemodiafiltration. Hemodialysis International. 2014; 18: 473-480. https://dx.doi.org/10.1111/hdi.12126. PMID: 24393428.

173. Meloni C, Ghezzi PM, Cipriani S, Petroni S, Tozzo C, Tatangelo P, et al. Hemodiafiltration with post-dilution reinfusion of the regenerated ultrafiltrate: a new on-line technique. Clinical Nephrology. 2005; 63: 106-112. PMID: 15730052.

174. Mercadal L, Franck JE, Metzger M, Torres PU, de Cornelissen F, Edet S, et al. Hemodiafiltration Versus Hemodialysis and Survival in Patients With ESRD: The French Renal Epidemiology and Information Network (REIN) Registry. American Journal of Kidney Diseases. 2016; 68: 247-255. 10.1053/j.ajkd.2015.11.016. PMID: WOS:000380753900016.

175. Mercadal L, Petitclerc T. [Technical advances in haemodialysis]. Nephrologie et Therapeutique. 2009; 5: 109-113. https://dx.doi.org/10.1016/j.nephro.2008.07.001. PMID: 19013119.

176. Mercadal L, Piekarski C, Renaux JL, Petitclerc T, Deray G. Isonatric dialysis biofeedback in hemodiafiltration with online regeneration of ultrafiltrate (HFR): rationale and study protocol for a randomized controlled study. Journal of Nephrology. 2012; 25: 1126-1130. https://dx.doi.org/10.5301/jn.5000084. PMID: 22322821.

177. Mesaros-Devcic I, Tomljanovic I, Mikolasevic I, Dvornik S, Vujicic B, Pavletic-Persic M, et al. Survival of patients treated with online hemodiafiltration compared to conventional hemodialysis. Collegium Antropologicum. 2013; 37: 827-832. PMID: 24308224.

178. Mesic E, Bock A, Major L, Vaslaki L, Berta K, Wikstroem B, et al. Maintaining high-efficiency on-line haemodiafiltration performances while reducing dialysis fluid consumption by automated coupling dialysate/blood flow functions: A European randomized cross-over study. NDT Plus. 2010; 3): iii154. PMID: 70483823.

179. Mesic E, Bock A, Major L, Vaslaki L, Berta K, Wikstrom B, et al. Dialysate saving by automated control of flow rates: comparison between individualized online hemodiafiltration and standard hemodialysis. Hemodialysis International. 2011; 15: 522-529. https://dx.doi.org/10.1111/j.1542-4758.2011.00577.x. PMID: 22111821.

180. Mineshima M, Eguchi K, Shishido K, Takahashi S, Kubo T, Kawaguchi H, et al. Clinical Effectiveness of Intermittent Infusion Hemodiafiltration Using Backfiltration of Ultrapure Dialysis Fluid Compared with Predilution On-Line Hemodiafiltration. Contributions to Nephrology. 2017; 189: 24-29. https://dx.doi.org/10.1159/000450636. PMID: 27951546.

181. Mitzner SR, Stange J, Klammt S, Risler T, Erley CM, Bader BD, et al. Improvement of hepatorenal syndrome with extracorporeal albumin dialysis MARS: results of a prospective, randomized, controlled clinical trial. Liver Transplantation. 2000; 6: 277-286. https://dx.doi.org/10.1002/lt.500060326. PMID: 10827226.

182. Molto J, Graterol F, Miranda C, Bancu I, Khoo S, Amara A, et al. Minimal removal of dolutegravir by hemodialysis in HIV-infected patients. Topics in Antiviral Medicine. 2016; 24 (E-1): 166. PMID: 613268736.

183. Mostovaya IM, Blankestijn PJ, Bots ML, Covic A, Davenport A, Grooteman MPC, et al. Clinical Evidence on Hemodiafiltration: A Systematic Review and a Meta- analysis. Seminars in Dialysis. 2014; 27: 119-127. 10.1111/sdi.12200. PMID: WOS:000332153300008.

184. Mostovaya IM, Blankestijn PJ, Grp C. What Have We Learned from CONTRAST? Blood Purification. 2013; 35: 39-44. 10.1159/000346376. PMID: WOS:000315556000009.

185. Mostovaya IM, Bots ML, van den Dorpel MA, Grooteman MP, Kamp O, Levesque R, et al. A randomized trial of hemodiafiltration and change in cardiovascular parameters. Clinical Journal of The American Society of Nephrology: CJASN. 2014; 9: 520-526. https://dx.doi.org/10.2215/CJN.07140713. PMID: 24408114.

186. Mostovaya IM, Grooteman MP, Basile C, Davenport A, de Roij van Zuijdewijn CL, Wanner C, et al. High convection volume in online post-dilution haemodiafiltration: relevance, safety and costs. Clinical Kidney Journal. 2015; 8: 368-373. https://dx.doi.org/10.1093/ckj/sfv040. PMID: 26251701.

187. Munoz R, Gallardo I, Valladares E, Saracho R, Martinez I, Ocharan J, et al. Online hemodiafiltration: 4 years of clinical experience. Hemodialysis International. 2006; 10 Suppl 1: S28-32. https://dx.doi.org/10.1111/j.1542-4758.2006.01187.x. PMID: 16441865.

188. Mustafa RA, Bdair F, Akl EA, Garg AX, Thiessen-Philbrook H, Salameh H, et al. Effect of Lowering the Dialysate Temperature in Chronic Hemodialysis: A Systematic Review and Meta-Analysis. Clinical Journal of the American Society of Nephrology. 2016; 11: 442-457. 10.2215/cjn.04580415. PMID: WOS:000371453100012.

189. Ng TG, Tan SH. Novel Trends in Haemodialysis: Where Are We Heading? Annals Academy of Medicine Singapore. 2010; 39: 482-488. PMID: WOS:000280032800013.

190. Nistor I, Palmer SC, Craig JC, Saglimbene V, Vecchio M, Covic A, et al. Convective Versus Diffusive Dialysis Therapies for Chronic Kidney Failure: An Updated Systematic Review of Randomized Controlled Trials. American Journal of Kidney Diseases. 2014; 63: 954-967. 10.1053/j.ajkd.2013.12.004. PMID: WOS:000336385900013.

191. Nistor I, Palmer SC, Craig JC, Saglimbene V, Vecchio M, Covic A, et al. Haemodiafiltration, haemofiltration and haemodialysis for end-stage kidney disease. Cochrane Database of Systematic Reviews. 2015: CD006258.pub006252. 10.1002/14651858.CD006258.pub2. PMID: WOS:000355993600016.

192. Noce A, Ferrannini M, Dessi M, Bocedi A, Fabrini R, Palumbo R, et al. Impact of hemodialytic procedures and dialytic doses on erythrocyte glutathione s-tranferase (E-GST) activity. International Journal of Artificial Organs. 2011; 34 (8): 672-673. http://dx.doi.org/10.5301/IJAO.2011.8702. PMID: 70717802.

193. Nube MJ, Peters SAE, Blankestijn PJ, Canaud B, Davenport A, Grooteman MPC, et al. Mortality reduction by post-dilution online-haemodiafiltration: A cause-specific analysis. Nephrology Dialysis Transplantation. 2017; 32: 548-555. http://dx.doi.org/10.1093/ndt/gfw381. PMID: 615290290.

194. Ok E, Asci G, Toz H, Ok ES, Kircelli F, Yilmaz M, et al. Mortality and cardiovascular events in online haemodiafiltration (OL-HDF) compared with high-flux dialysis: results from the Turkish OL-HDF Study. Nephrology Dialysis Transplantation. 2013; 28: 192-202. https://dx.doi.org/10.1093/ndt/gfs407. PMID: 23229932.

195. Okano K, Tsuchiya K, Nitta K. Removal efficiency of protein bound solutes and antioxidants by online hemodiafiltration compared with high-flux hemodialysis. Nephrology Dialysis Transplantation. 2016; 1): i231-i232. http://dx.doi.org/10.1093/ndt/gfw170.28. PMID: 72326523.

196. Ortiz A, Covic A, Fliser D, Fouque D, Goldsmith D, Kanbay M, et al. Epidemiology, contributors to, and clinical trials of mortality risk in chronic kidney failure. Lancet. 2014; 383: 1831-1843. 10.1016/s0140-6736(14)60384-6. PMID: WOS:000336267800030.

197. Palleschi S, Ghezzi PM, Palladino G, Rossi B, Ganadu M, Casu D, et al. Vitamins (A, C and E) and oxidative status of hemodialysis patients treated with HFR and HFR-Supra. BMC Nephrology. 2016; 17: 120. https://dx.doi.org/10.1186/s12882-016-0315-6. PMID: 27566671.

198. Panichi V, De Ferrari G, Saffioti S, Sidoti A, Biagioli M, Bianchi S, et al. Divert to ULTRA: differences in infused volumes and clearance in two on-line hemodiafiltration treatments. International Journal of Artificial Organs. 2012; 35: 435-443. https://dx.doi.org/10.5301/ijao.5000106. PMID: 22669588.

199. Panichi V, Fiaccadori E, Rosati A, Fanelli R, Bernabini G, Scatena A, et al. Post-dilution on line haemodiafiltration with citrate dialysate: first clinical experience in chronic dialysis patients. Thescientificworldjournal. 2013; 2013: 703612. https://dx.doi.org/10.1155/2013/703612. PMID: 24367243.

200. Panichi V, Manca-Rizza G, Paoletti S, Taccola D, Consani C, Filippi C, et al. Effects on inflammatory and nutritional markers of haemodiafiltration with online regeneration of ultrafiltrate (HFR) vs online haemodiafiltration: a cross-over randomized multicentre trial. Nephrology Dialysis Transplantation. 2006; 21: 756-762. https://dx.doi.org/10.1093/ndt/gfi189. PMID: 16303780.

201. Panichi V, Rizza GM, Paoletti S, Bigazzi R, Aloisi M, Barsotti G, et al. Chronic inflammation and mortality in haemodialysis: effect of different renal replacement therapies. Results from the RISCAVID study. Nephrology Dialysis Transplantation. 2008; 23: 2337-2343. https://dx.doi.org/10.1093/ndt/gfm951. PMID: 18305316.

202. Panichi V, Scatena A, Rosati A, Giusti R, Ferro G, Malagnino E, et al. High-volume online haemodiafiltration improves erythropoiesis-stimulating agent (ESA) resistance in comparison with low-flux bicarbonate dialysis: results of the REDERT study. Nephrology Dialysis Transplantation. 2015; 30: 682-689. https://dx.doi.org/10.1093/ndt/gfu345. PMID: 25385719.

203. Pedrini L. Preliminary results of high-efficiency on-line mixed hemodiafiltration in a dialysis centre network in italy (nephrocare). Nephrology Dialysis Transplantation. 2013; 1): i417-i418. http://dx.doi.org/10.1093/ndt/gft144. PMID: 71076288.

204. Pedrini L. Mixed haemodiafiltration. Long-term effects on efficiency and survival. A cohort study. Nephrology Dialysis Transplantation. 2017; 32 (Supplement 3): iii279-iii280. http://dx.doi.org/10.1093/ndt/gfx150. PMID: 617290200.

205. Pedrini L, Mari F, Barbieri C, Cattinelli I, Bellocchio F, Amato C. Determinants of beta-2 microglobulin (beta2-M) levels in patients on long-term haemodiafiltration (HDF). Computational technique analysis of the records from a large data-base system (EUCLID). Nephrology Dialysis Transplantation. 2013; 1): i31. http://dx.doi.org/10.1093/ndt/gft159. PMID: 71075099.

206. Pedrini LA, Cozzi G, Faranna P, Mercieri A, Ruggiero P, Zerbi S, et al. Transmembrane pressure modulation in high-volume mixed hemodiafiltration to optimize efficiency and minimize protein loss. Kidney International. 2006; 69: 573-579. https://dx.doi.org/10.1038/sj.ki.5000110. PMID: 16407883.

207. Pedrini LA, De Cristofaro V. On-line mixed hemodiafiltration with a feedback for ultrafiltration control: effect on middle-molecule removal. Kidney International. 2003; 64: 1505-1513. https://dx.doi.org/10.1046/j.1523-1755.2003.00240.x. PMID: 12969172.

208. Pedrini LA, De Cristofaro V, Comelli M, Casino FG, Prencipe M, Baroni A, et al. Long-term effects of high-efficiency on-line haemodiafiltration on uraemic toxicity. A multicentre prospective randomized study. Nephrology Dialysis Transplantation. 2011; 26: 2617-2624. https://dx.doi.org/10.1093/ndt/gfq761. PMID: 21245130.

209. Pedrini LA, Feliciani A, Zerbi S, Cozzi G, Ruggiero P. Optimization of mid-dilution haemodiafiltration: technique and performance. Nephrology Dialysis Transplantation. 2009; 24: 2816-2824. https://dx.doi.org/10.1093/ndt/gfp207. PMID: 19420103.

210. Pedrini LA, Gmerek A, Wagner J. Efficiency of post-dilution hemodiafiltration with a high-flux alpha-polysulfone dialyzer. International Journal of Artificial Organs. 2011; 34: 397-404. https://dx.doi.org/10.5301/IJAO.2011.8330. PMID: 21574157.

211. Pedrini LA, Wiesen G. Overcoming the limitations of post-dilution on-line hemodiafiltration: mixed dilution hemodiafiltration. Contributions to Nephrology. 2011; 175: 129-140. https://dx.doi.org/10.1159/000333629. PMID: 22188695.

212. Pellicano R, Polkinghorne KR, Kerr PG. Reduction in beta(2)-microglobulin with super-flux versus high-flux dialysis membranes: Results of a 6-week, randomized, double-blind, crossover trial. American Journal of Kidney Diseases. 2008; 52: 93-101. 10.1053/j.ajkd.2008.02.296. PMID: WOS:000257886600013.

213. Penne EL, Blankestijn PJ, Bots ML, van den Dorpel MA, Grooteman MP, Nube MJ, et al. [New study evaluating online haemodiafiltration for the reduction of cardiovascular morbidity and mortality in patients undergoing chronic haemodialysis]. Nederlands Tijdschrift voor Geneeskunde. 2006; 150: 1583-1585. PMID: 16886698.

214. Penne EL, Blankestijn PJ, Bots ML, van den Dorpel MA, Grooteman MP, Nube MJ, et al. Effect of increased convective clearance by on-line hemodiafiltration on all cause and cardiovascular mortality in chronic hemodialysis patients - The Dutch CONvective TRAnsport STudy (CONTRAST): Rationale and design of a randomised controlled trial [ISRCTN38365125]. Current controlled trials in cardiovascular medicine. 2005; 6: 10. PMID: CN-00557388 UPDATE.

215. Penne EL, Blankestijn PJ, Bots ML, van den Dorpel MA, Grooteman MP, Nube MJ, et al. Effect of increased convective clearance by on-line hemodiafiltration on all cause and cardiovascular mortality in chronic hemodialysis patients - the Dutch CONvective TRAnsport STudy (CONTRAST): rationale and design of a randomised controlled trial [ISRCTN38365125]. Current Controlled Trials in Cardiovascular Medicine. 2005; 6: 8. https://dx.doi.org/10.1186/1468-6708-6-8. PMID: 15907201.

216. Penne EL, Blankestijn PJ, Bots ML, van den Dorpel MA, Grooteman MPC, Nube MJ, et al. Resolving controversies regarding hemodiafiltration versus hemodialysis: The Dutch Convective Transport Study. Seminars in Dialysis. 2005; 18: 47-51. 10.1111/j.1525-139X.2005.18107.x. PMID: WOS:000226440500014.

217. Penne EL, van der Weerd NC, Blankestijn PJ, van den Dorpel MA, Grooteman MP, Nube MJ, et al. Role of residual kidney function and convective volume on change in beta2-microglobulin levels in hemodiafiltration patients. Clinical Journal of The American Society of Nephrology: CJASN. 2010; 5: 80-86. https://dx.doi.org/10.2215/CJN.03340509. PMID: 19965537.

218. Penne EL, van der Weerd NC, van den Dorpel MA, Grooteman MP, Levesque R, Nube MJ, et al. Short-term effects of online hemodiafiltration on phosphate control: a result from the randomized controlled Convective Transport Study (CONTRAST). American Journal of Kidney Diseases. 2010; 55: 77-87. https://dx.doi.org/10.1053/j.ajkd.2009.09.023. PMID: 19962805.

219. Penne EL, van der Weerd NC, van den Dorpel MA, Grooteman MPC, Levesque R, Nube MJ, et al. Short-term Effects of Online Hemodiafiltration on Phosphate Control: A Result From the Randomized Controlled Convective Transport Study (CONTRAST). American Journal of Kidney Diseases. 2010; 55: 77-87. 10.1053/j.ajkd.2009.09.023. PMID: WOS:000273958000012.

220. Perez-Garcia R, Albalate M, de Sequera P, Alcazar R, Puerta M, Ortega M, et al. On-line haemodiafiltration improves response to calcifediol treatment. Nefrologia. 2012; 32: 459-466. https://dx.doi.org/10.3265/Nefrologia.pre2012.Jan.11189. PMID: 22652556.

221. Peters S, Bots M, Canaud B, Davenport A, Grooteman M, Kircelli F, et al. Haemodiafiltration and mortality in end stage kidney disease patients an individual participant data meta-analysis of randomised controlled trials. Nephrology Dialysis Transplantation. 2015; 30: iii306. PMID: CN-01139470 NEW.

222. Peters SA, Bots ML, Canaud B, Davenport A, Grooteman MP, Kircelli F, et al. Haemodiafiltration and mortality in end-stage kidney disease patients: a pooled individual participant data analysis from four randomized controlled trials. Nephrology Dialysis Transplantation. 2016; 31: 978-984. https://dx.doi.org/10.1093/ndt/gfv349. PMID: 26492924.

223. Petras D, Fortunato A, Soffiati G, Brendolan A, Bonello M, Crepaldi C, et al. Sequential convective therapies (SCT): a prospective study on feasibility, safety, adequacy and tolerance of on-line hemofiltration and hemodiafiltration in sequence. International Journal of Artificial Organs. 2005; 28: 482-488. PMID: 15883963.

224. Petrie JJB, Ng TG, Hawley CM. Review Article: Is it time to embrace haemodiafiltration for centre-based haemodialysis? Nephrology. 2008; 13: 269-277. 10.1111/j.1440-1797.2008.00964.x. PMID: WOS:000256701100001.

225. Pettila V, Tiula E. Intermittent hemodiafiltration in acute renal failure in critically ill patients. Clinical Nephrology. 2001; 56: 324-331. PMID: 11680663.

226. Pizzarelli F. Is online hemodiafiltration associated with greater cardiovascular stability than hemodialysis? Commentary. Nature Clinical Practice Nephrology. 2006; 2: 480-481. http://dx.doi.org/10.1038/ncpneph0251. PMID: 44325956.

227. Pizzarelli F, Cerrai T, Dattolo P, Ferro G. On-line haemodiafiltration with and without acetate. Nephrology Dialysis Transplantation. 2006; 21: 1648-1651. https://dx.doi.org/10.1093/ndt/gfk093. PMID: 16464887.

228. Pizzarelli F, Cerrai T, Dattolo P, Tetta C, Maggiore Q. Convective treatments with on-line production of replacement fluid: a clinical experience lasting 6 years. Nephrology Dialysis Transplantation. 1998; 13: 363-369. PMID: 9509447.

229. Ponce P, Chung T, Kreuzberg U. Fluid management in hemodialysis: Conventional clinical management vs. body composition monitoring (BCM) supported management of overhydrated patients. Nephrology Dialysis Transplantation. 2013; 1): i417. http://dx.doi.org/10.1093/ndt/gft144.

230. Potier J, Le Roy F, Faucon JP, Besselievre T, Renaudineau E, Farquet C, et al. Elevated removal of middle molecules without significant albumin loss with mixed-dilution hemodiafiltration for patients unable to provide sufficient blood flow rates. Blood Purification. 2013; 36: 78-83. https://dx.doi.org/10.1159/000351527. PMID: 23989087.

231. Potier J, Queffeulou G, Bouet J. Are all dialyzers compatible with the convective volumes suggested for postdilution online hemodiafiltration? International Journal of Artificial Organs. 2016; 39: 460-470. https://dx.doi.org/10.5301/ijao.5000525. PMID: 27791259.

232. Power A, Ashby D. Haemodialysis: hospital or home? Postgraduate Medical Journal. 2014; 90: 92-97. 10.1136/postgradmedj-2012-131405. PMID: WOS:000337784800007.

233. Rabindranath KS, Strippoli GFM, Roderick P, Wallace SA, MacLeod AM, Daly C. Comparison of hemodialysis, hemofiltration, and acetate-free biofiltration for ESRD: Systematic review. American Journal of Kidney Diseases. 2005; 45: 437-447. 10.1053/j.ajkd.2004.11.008. PMID: WOS:000227873000001.

234. Ragazzoni E, Carpani P, Agliata S, Ciranna G, Cusinato S, Albini M, et al. [HFR vs HDF-ON line: plasmatic amino acids loss evaluation]. Giornale Italiano di Nefrologia. 2004; 21 Suppl 30: S85-90. PMID: 15747313.

235. Ramponi F, Ronco C, Mason G, Rettore E, Marcelli D, Martino F, et al. Cost-effectiveness analysis of online hemodiafiltration versus high-flux hemodialysis. Clinicoeconomics & Outcomes Research. 2016; 8: 531-540. https://dx.doi.org/10.2147/CEOR.S109649. PMID: 27703388.

236. Righetti M, Ferrario GM, Milani S, Serbelloni P, Sessa A. [A single centre study about the effects of HFR on anemia]. Giornale Italiano di Nefrologia. 2004; 21 Suppl 30: S168-171. PMID: 15750978.

237. Righetti M, Filiberti O, Ranghino A, Ferrario G, Milani S, Serbelloni P, et al. Internal hemodiafiltration versus low-flux bicarbonate dialysis: Results from a long-term prospective study. International Journal of Artificial Organs. 2010; 33: 796-802. PMID: 21140355.

238. Ronco C. Hemodiafiltration: Evolution of a technique towards better dialysis care. Contributions to Nephrology. 2011; 168: 19-27. https://dx.doi.org/10.1159/000321741. PMID: 20938122.

239. Ronco C, Cruz D. Hemodiafiltration history, technology, and clinical results. Advances in Chronic Kidney Disease. 2007; 14: 231-243. https://dx.doi.org/10.1053/j.ackd.2007.04.002. PMID: 17603976.

240. Roumelioti ME, Nolin T, Unruh ML, Argyropoulos C. Revisiting the Middle Molecule Hypothesis of Uremic Toxicity: A Systematic Review of Beta 2 Microglobulin Population Kinetics and Large Scale Modeling of Hemodialysis Trials In Silico. PLoS ONE [Electronic Resource]. 2016; 11: e0153157. https://dx.doi.org/10.1371/journal.pone.0153157. PMID: 27055286.

241. Samtleben W, Dengler C, Reinhardt B, Nothdurft A, Lemke HD. Comparison of the new polyethersulfone high-flux membrane DIAPES HF800 with conventional high-flux membranes during on-line haemodiafiltration. Nephrology Dialysis Transplantation. 2003; 18: 2382-2386. PMID: 14551370.

242. Santoro A. Online HDF -mechanisms of solute and fluid removal in haemodiafiltration. Nephrology. 2010; 3): 25. http://dx.doi.org/10.1111/j.1440-1797.2010.01335.x. PMID: 70467331.

243. Sawires H, Makar S, Zekry H. Effect of On-Line Hemodiafiltration on P-Wave Dispersion in Children. Therapeutic Apheresis & Dialysis: Official Peer-Reviewed Journal of the International Society for Apheresis, the Japanese Society for Apheresis, the Japanese Society for Dialysis Therapy. 2015; 19: 399-404. https://dx.doi.org/10.1111/1744-9987.12285. PMID: 25810248.

244. Schindler R. Hemodialysis: What is new?. [German]. Nephrologe. 2016; 11: 312-319. http://dx.doi.org/10.1007/s11560-016-0070-5. PMID: 610775281.

245. Schindler R, Stahl C, Venz S, Ludat K, Krause W, Frei U. Removal of contrast media by different extracorporeal treatments. Nephrology Dialysis Transplantation. 2001; 16: 1471-1474. PMID: 11427643.

246. Schmid H, Schiffl H. Hemodiafiltration and survival of end-stage renal disease patients: the long journey goes on. International Urology & Nephrology. 2012; 44: 1435-1440. https://dx.doi.org/10.1007/s11255-012-0232-y. PMID: 22752499.

247. Selby NM, Fluck RJ, Taal MW, McIntyre CW. Effects of acetate-free double-chamber hemodiafiltration and standard dialysis on systemic hemodynamics and troponin T levels. ASAIO Journal. 2006; 52: 62-69. https://dx.doi.org/10.1097/01.mat.0000189725.93808.58. PMID: 16436892.

248. Siriopol D, Canaud B, Stuard S, Mircescu G, Nistor I, Covic A. New insights into the effect of haemodiafiltration on mortality: the Romanian experience. Nephrology Dialysis Transplantation. 2015; 30: 294-301. 10.1093/ndt/gfu347. PMID: WOS:000351660000022.

249. Sirolli V, Cappelli P, Amoroso L, Di Liberato L, Muscianese P, Santarelli P, et al. [On-line HFR and removal of uremic toxins inducing the loss of phospholipidic asymmetry of the erythrocyte membrane]. Giornale Italiano di Nefrologia. 2004; 21 Suppl 30: S208-211. PMID: 15750987.

250. Skofic N, Arnol M, Buturovic-Ponikvar J, Ponikvar R. Intermittent high-volume predilution on-line haemofiltration versus standard intermittent haemodialysis in critically ill patients with acute kidney injury: a prospective randomized study. Nephrology Dialysis Transplantation. 2012; 27: 4348-4356. https://dx.doi.org/10.1093/ndt/gfs068. PMID: 22513706.

251. Susantitaphong P, Siribamrungwong M, Jaber BL. Convective therapies versus low-flux hemodialysis for chronic kidney failure: a meta-analysis of randomized controlled trials. Nephrology Dialysis Transplantation. 2013; 28: 2859-2874. 10.1093/ndt/gft396. PMID: WOS:000326747600033.

252. Susantitaphong P, Tiranathanagul K, Katavetin P, Hanwiwatwong O, Wittayalertpanya S, Praditpornsilpa K, et al. Efficacy comparison between simple mixed-dilution and simple mid-dilution on-line hemodiafiltration techniques: a crossover study. Artificial Organs. 2012; 36: 1059-1065. https://dx.doi.org/10.1111/j.1525-1594.2012.01508.x. PMID: 22882579.

253. Susantitaphong P, Tiranathanagul K, Katavetin P, Townamchai N, Praditpornsilpa K, Tungsanga K, et al. Efficacy of convective-controlled double high-flux hemodiafiltration versus on-line hemodiafiltration: 1-year prospective study. Blood Purification. 2010; 29: 35-43. https://dx.doi.org/10.1159/000255955. PMID: 19907162.

254. Teatini U, Steckiph D, Romei Longhena G. Evaluation of a new online hemodiafiltration mode with automated pressure control of convection. Blood Purification. 2011; 31: 259-267. https://dx.doi.org/10.1159/000321884. PMID: 21242679.

255. Tharapath R, Trakarnvanich T, Prommool S, Teeprasan T, Kurathong S. Quality of life in end stage kidney disease patients: A comparison between conventional hemodialysis and on-line hemodiafiltration. Nephrology. 2010; 3): 112-113. http://dx.doi.org/10.1111/j.1440-1797.2010.01337.x. PMID: 70467664.

256. Thumfart J, Pommer W, Querfeld U, Muller D. Intensified Hemodialysis in Adults, and in Children and Adolescents. Deutsches Arzteblatt International. 2014; 111: 237-243. 10.3238/arztebl.2014.0237. PMID: WOS:000334598200001.

257. Tiranathanagul K, Tangvoraphonkchai K, Srisawat N, Susantitaphong P, Tungsanga K, Praditpornsilpa K, et al. Acute intradialytic cardiac function and inflammatory cytokine changes during high-efficiency online hemodiafiltration with acetate-free and standard dialysis solutions. Therapeutic Apheresis & Dialysis: Official Peer-Reviewed Journal of the International Society for Apheresis, the Japanese Society for Apheresis, the Japanese Society for Dialysis Therapy. 2015; 19: 250-258. https://dx.doi.org/10.1111/1744-9987.12271. PMID: 25545821.

258. Tiranathanagul K, Yossundharakul C, Techawathanawanna N, Katavetin P, Hanvivatvong O, Praditpornsilp K, et al. Comparison of middle-molecule clearance between convective control double high-flux hemodiafiltration and on-line hemodiafiltration. International Journal of Artificial Organs. 2007; 30: 1090-1097. PMID: 18203071.

259. Tomo T, Matsuyama K, Nasu M. Effect of hemodiafiltration against radical stress in the course of blood purification. Blood Purification. 2004; 22 Suppl 2: 72-77. https://dx.doi.org/10.1159/000081879. PMID: 15655328.

260. Tomo T, Shinoda T. Biocompatibility of dialysis fluid for online HDF. Contributions to Nephrology. 2011; 168: 89-98. https://dx.doi.org/10.1159/000321747. PMID: 20938128.

261. Tsobaneli J, Tsobanelis T, Kurz P, Hensel N, Obermann K, Schwenger V. Health-related quality of life in high convective volume online hemodiafiltration. Nephrology Dialysis Transplantation. 2014; 29: iii282. PMID: CN-01010190 UPDATE.

262. Tsuchida K, Minakuchi J. Clinical benefits of predilution on-line hemodiafiltration. Blood Purification. 2013; 35 Suppl 1: 18-22. https://dx.doi.org/10.1159/000346221. PMID: 23466373.

263. Tuaillon E, Jaussent I, Morena M, Rodrigez A, Chenine L, Kuster N, et al. T-Cell Activation and Malnutrition Adversely Impact on Endothelial Progenitor Cell Mobilization in Patients on Extracorporeal Maintenance Dialysis Therapy. Blood Purification. 2015; 39: 313-322. https://dx.doi.org/10.1159/000381661. PMID: 25998198.

264. Van Buren PN, Inrig JK. Hypertension and hemodialysis: pathophysiology and outcomes in adult and pediatric populations. Pediatric Nephrology. 2012; 27: 339-350. 10.1007/s00467-011-1775-3. PMID: WOS:000299506100001.

265. van der Weerd NC, Den Hoedt CH, Blankestijn PJ, Bots ML, van den Dorpel MA, Levesque R, et al. Resistance to erythropoiesis stimulating agents in patients treated with online hemodiafiltration and ultrapure low-flux hemodialysis: results from a randomized controlled trial (CONTRAST). PLoS ONE [Electronic Resource]. 2014; 9: e94434. https://dx.doi.org/10.1371/journal.pone.0094434. PMID: 24743493.

266. van Zuijdewijn C, Grooteman MPC, Bots ML, Blankestijn PJ, Steppan S, Buechel J, et al. Serum Magnesium and Sudden Death in European Hemodialysis Patients. Plos One. 2015; 10: e0143104. 10.1371/journal.pone.0143104. PMID: WOS:000365853900065.

267. Vanholder R, Glorieux G, Van Biesen W. Advantages of New Hemodialysis Membranes and Equipment. Nephron Clinical Practice. 2010; 114: C165-C172. 10.1159/000262298. PMID: WOS:000274696300002.

268. Vaslaki L, Major L, Berta K, Karatson A, Misz M, Pethoe F, et al. On-line haemodiafiltration versus haemodialysis: stable haematocrit with less erythropoietin and improvement of other relevant blood parameters. Blood Purification. 2006; 24: 163-173. https://dx.doi.org/10.1159/000090117. PMID: 16352871.

269. Vaslaki LR, Berta K, Major L, Weber V, Weber C, Wojke R, et al. On-line hemodiafiltration does not induce inflammatory response in end-stage renal disease patients: results from a multicenter cross-over study. Artificial Organs. 2005; 29: 406-412. https://dx.doi.org/10.1111/j.1525-1594.2005.29068.x. PMID: 15854217.

270. Velasco N. Convection with conviction-online hemodiafiltration for all: single center clinical observations. Hemodialysis International. 2006; 10 Suppl 1: S67-71. https://dx.doi.org/10.1111/j.1542-4758.2006.01195.x. PMID: 16441874.

271. Viegas M, Candido C, Felgueiras J, Clemente J, Barros S, Farbota R, et al. Dialysate bicarbonate variation in patients on regular hemodiafiltration program: Impact on intradialytic hypotension and interdialytic weight gain. Nephrology Dialysis Transplantation. 2016; 1): i520. http://dx.doi.org/10.1093/ndt/gfw196.18. PMID: 72327390.

272. Vilar E, Farrington K, Bates C, Mumford C, Greenwood R. Optimizing Home Dialysis: Role of Hemodiafiltration. In: Kawanishi H, Yamashita AC, editors. Hemodiafiltration: A New Era. 168. 2011. pp. 64-77.

273. Vilar E, Fry AC, Wellsted D, Tattersall JE, Greenwood RN, Farrington K. Long-term outcomes in online hemodiafiltration and high-flux hemodialysis: a comparative analysis. Clinical Journal of The American Society of Nephrology: CJASN. 2009; 4: 1944-1953. https://dx.doi.org/10.2215/CJN.05560809. PMID: 19820129.

274. Wagner S, Rode C, Erlenkoetter A, Brand K, Kreuzberg U, Gauly A. Comparative biocompatibility of polysulfone hemodialysers applied in different treatment modalities. Nephrology Dialysis Transplantation. 2017; 32 (Supplement 3): iii615. http://dx.doi.org/10.1093/ndt/gfx175. PMID: 617302515.

275. Wang AY, Ninomiya T, Al-Kahwa A, Perkovic V, Gallagher MP, Hawley C, et al. Effect of Hemodiafiltration or Hemofiltration Compared With Hemodialysis on Mortality and Cardiovascular Disease in Chronic Kidney Failure: A Systematic Review and Meta-analysis of Randomized Trials. American Journal of Kidney Diseases. 2014; 63: 968-978. 10.1053/j.ajkd.2014.01.435. PMID: WOS:000336385900014.

276. Ward RA. Protein-leaking membranes for hemodialysis: A new class of membranes in search of an application? Journal of the American Society of Nephrology. 2005; 16: 2421-2430. 10.1681/asn.2005010070. PMID: WOS:000230774700021.

277. Weber C, Stummvoll HK, Passon S, Falkenhagen D. Monocyte activation and humoral immune response to endotoxins in patients receiving on-line hemodiafiltration therapy. International Journal of Artificial Organs. 1998; 21: 335-340. PMID: 9714027.

278. Weinreich T. New developments in dialysis. [German]. Nephrologe. 2014; 9: 139-142. http://dx.doi.org/10.1007/s11560-013-0819-z. PMID: 53001964.

279. Wizemann V. Low- (classical) and high-efficiency haemodiafiltration. Contributions to Nephrology. 2007; 158: 103-109. https://dx.doi.org/10.1159/0000107240. PMID: 17684348.

280. Wizemann V, Lotz C, Techert F, Uthoff S. On-line haemodiafiltration versus low-flux haemodialysis. A prospective randomized study. Nephrology Dialysis Transplantation. 2000; 15 Suppl 1: 43-48. PMID: 10737166.

281. Yilmaz A, Yildirim ZY, Gedikbasi A, Aksu B, Agbas A, Pehlivanoglu C, et al. Vascular endothelial dysfunction in renal replacement therapy modalities. Pediatric Nephrology. 2017; 32 (9): 1717-1718. http://dx.doi.org/10.1007/s00467-017-3753-x. PMID: 618119507.

282. Yu X. The Evolving Patterns of Uremia: Unmet Clinical Needs in Dialysis. Contributions to Nephrology. 2017; 191: 1-7. https://dx.doi.org/10.1159/000479251. PMID: 28910786.

283. Zawada AM, Michel AI, Emrich IE, Seiler-Musler S, Van Bentum K, Boslet R, et al. Online hemodiafiltration eliminates's adenosylhomocysteine more efficiently than standard hemodialysis. Nephrology Dialysis Transplantation. 2016; 31: i521. PMID: CN-01167635 NEW.

284. Zhang W, Mei C, Chen N, Chen X, Zou J, Xue J, et al. Improving adequacy of hemodialysis in Shanghai: Perspectives from the quality control group of the Shanghai renal registry network (SRRN). Medical Science Technology. 2015; 56: 78-83. http://dx.doi.org/10.12659/MST.895556. PMID: 614907698.

285. Zickler D, Schindler R, Willy K, Martus P, Pawlak M, Storr M, et al. Medium Cut-Off (MCO) Membranes Reduce Inflammation in Chronic Dialysis Patients-A Randomized Controlled Clinical Trial. Plos One. 2017; 12: e0169024. 10.1371/journal.pone.0169024. PMID: WOS:000391972600011.
