# Supplementary material for: Effect of online hemodiafiltration compared with hemodialysis on quality of life in patients with ESRD: A systematic review and meta-analysis of randomized trials
Source: PLoS One. 2018 Oct 18;13(10):e0205037. doi: 10.1371/journal.pone.0205037 (PMC6193628; doi:10.1371/journal.pone.0205037)
Supplement: S6 Appendix — (DOC) [file pone.0205037.s006.doc]

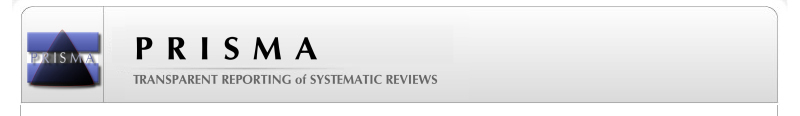
**PRISMA 2009 Flow Diagram**

**Screening**

**Included**

**Eligibility**

**Identification**

Records identified through database searching
(n = 1264)

Additional records identified through other sources
(n = 564)

Records after duplicates removed
(n = 292)

Records screened
(n = 292)

Records excluded (n = 242)

-Not original research (n = 71)

-Not RCT (n= 35)

-Not relevant (n= 136)

Full-text articles assessed for eligibility
(n = 50)

Full-text articles excluded, with reasons (n = 44)

-Not original research (n= 2)

-Conference report (n= 2)

-Not relevant (n= 40)

Studies included in qualitative synthesis
(n = 7)

Studies included in quantitative synthesis (meta-analysis)
(n = 6)

Full-text articles excluded, with reasons (n = 1)

-Not common scales of QoL with other RCTs included (n= 1)
